# Human milk oligosaccharide 2’-fucosyllactose protects against high-fat diet-induced obesity by changing intestinal mucus production, composition and degradation linked to changes in gut microbiota and faecal proteome profiles in mice

**DOI:** 10.1136/gutjnl-2023-330301

**Published:** 2024-05-13

**Authors:** Paola Paone, Dimitris Latousakis, Romano Terrasi, Didier Vertommen, Ching Jian, Valentina Borlandelli, Francesco Suriano, Malin E V Johansson, Anthony Puel, Caroline Bouzin, Nathalie M Delzenne, Anne Salonen, Nathalie Juge, Bogdan I Florea, Giulio G Muccioli, Herman Overkleeft, Matthias Van Hul, Patrice D Cani

**Affiliations:** 1 Louvain Drug Research Institute (LDRI), Metabolism and Nutrition research group (MNUT), UCLouvain, Université catholique de Louvain, Brussels, Belgium; 2 The Gut Microbiome and Health and Food Safety Institute Strategic Programme, Norwich Research Park, Quadram Institute Bioscience, Norwich, UK; 3 Louvain Drug Research Institute (LDRI), Bioanalysis and Pharmacology of Bioactive Lipids Research Group (BPBL), UCLouvain, Université catholique de Louvain, Brussels, Belgium; 4 de Duve Institute, MASSPROT platform, UCLouvain, Université catholique de Louvain, Brussels, Belgium; 5 Human Microbiome Research Program, Faculty of Medicine, University of Helsinki, Helsinki, Finland; 6 Department Bio-organic Synthesis, Leids Instituut voor Chemisch Onderzoek, Leiden University, Leiden, The Netherlands; 7 Department of Medical Biochemistry and Cell Biology, Institute of Biomedicine, University of Gothenburg, Gothenburg, Sweden; 8 Walloon Excellence in Life Sciences and BIOtechnology (WELBIO) Department, WEL Research Institute, Wavre, Belgium; 9 Institute of Experimental and Clinical Research (IREC), IREC Imaging Platform (2IP RRID:SCR_023378), UCLouvain, Université catholique de Louvain, Brussels, Belgium; 10 Institute of Experimental and Clinical Research (IREC), UCLouvain, Université catholique de Louvain, Brussels, Belgium

**Keywords:** MUCUS, OBESITY, INTESTINAL MICROBIOLOGY, MUCOSAL BARRIER, PREBIOTIC

## Abstract

**Objective:**

To decipher the mechanisms by which the major human milk oligosaccharide (HMO), 2’-fucosyllactose (2’FL), can affect body weight and fat mass gain on high-fat diet (HFD) feeding in mice. We wanted to elucidate whether 2’FL metabolic effects are linked with changes in intestinal mucus production and secretion, mucin glycosylation and degradation, as well as with the modulation of the gut microbiota, faecal proteome and endocannabinoid (eCB) system.

**Results:**

2’FL supplementation reduced HFD-induced obesity and glucose intolerance. These effects were accompanied by several changes in the intestinal mucus layer, including mucus production and composition, and gene expression of secreted and transmembrane mucins, glycosyltransferases and genes involved in mucus secretion. In addition, 2’FL increased bacterial glycosyl hydrolases involved in mucin glycan degradation. These changes were linked to a significant increase and predominance of bacterial genera *Akkermansia* and *Bacteroides*, different faecal proteome profile (with an upregulation of proteins involved in carbon, amino acids and fat metabolism and a downregulation of proteins involved in protein digestion and absorption) and, finally, to changes in the eCB system. We also investigated faecal proteomes from lean and obese humans and found similar changes observed comparing lean and obese mice.

**Conclusion:**

Our results show that the HMO 2’FL influences host metabolism by modulating the mucus layer, gut microbiota and eCB system and propose the mucus layer as a new potential target for the prevention of obesity and related disorders.

**WHAT IS ALREADY KNOWN ON THIS TOPIC**

- High-fat diet-induced obesity and metabolic disorders is associated with alterations in microbiota profile and gut barrier function.
- The intestinal mucus layer is altered during high-fat diet (HFD), western-style diet, low-fibre diet, emulsifier treatments and in genetically obese (*ob/ob*) mice and human with dysglycaemia. The alterations observed include increased penetrability, decreased thickness, reduced growth and different mucin glycans composition.
- Prebiotic treatments such as 2’-fucosyllactose (2’FL) improved gut barrier integrity in an in vitro model by affecting the mucus layer, but no studies have investigated whether the mucus is involved in the protection against obesity in vivo.

**WHAT THIS STUDY ADDS**

- This study shows that supplementing 2’FL to HFD reduces the increase in body weight and fat mass, attenuates glucose intolerance and affects hormones involved in appetite regulation and energy homeostasis.
- 2’FL supplementation affects the mucus layer in vivo in the context of obesity, by increasing mucus production, secreted and transmembrane mucins, glycosyltransferases and glycosyl hydrolases, and mucin glycosylation.
- Bacterial communities in mice fed with HFD plus 2’FL are remarkably enriched in *Akkermansia* and *Bacteroides* genera.
- 2’FL supplementation changes faecal proteome profiles, increasing proteins involved in carbon, amino acids and fat metabolism and decreasing those involved in protein digestion and absorption.
- Supplementing 2’FL affects the intestinal endocannabinoid system.

**HOW THIS STUDY MIGHT AFFECT RESEARCH, PRACTICE OR POLICY**

- 2‘FL is a prebiotic found naturally in the breast milk of about 80% of mothers. Excluding water, human milk oligosaccharides are the third most abundant ingredient in breast milk after fat and carbohydrates. Understanding their mechanism of actions and effects is vital information.
- There is increased interest in 2’FL to be used as a supplement, not only in infant formula but also for subjects with 2’FL synthesis deficiency (ie, *Fut2* genetic polymorphisms inducing fucosyltransferase inactivity).
- This study shows that the mechanisms by which 2’FL counteracts obesity and metabolic disorders are associated with changes in the intestinal mucus layer and points towards the mucus as a new potential therapeutic target for the prevention and/or treatment of obesity and metabolic disorders.

## Introduction

Obesity is associated with several metabolic alterations like type 2 diabetes, cardiovascular diseases and changes in the gut microbiota composition and gut barrier disruption.^1^ Among the components of the gut barrier, it has been shown that the mucus layer is altered when the mice are fed a high-fat diet (HFD), western-style diet (WSD) or low-fibre diet and in *ob/ob* mice, as well as in patients with dysglycaemia. Among the alterations, it has been observed a reduced thickness, increased penetrability and altered mucin glycan composition.^2–9^ The mucus exerts important roles in gut barrier protection and represents the interface of communication between bacteria and host. It is produced by the goblet cells (GCs) and constituted of glycoproteins called mucins, among which the main component is the secreted Muc2. The transmembrane mucins, involved in glycocalyx formation, are other important components of the gut barrier, conferring cell protection and mediating host–microbe interactions.^10^ Mucins are glycosylated thanks to glycosyltransferases and mucin glycans supply attachment sites and allow bacterial growth and colonisation. Indeed, bacteria are able to produce glycosyl hydrolases (GHs) to degrade mucin glycans and use them as energy source.^10^

α−1,2-fucosyltransferase, encoded by the *FUT2* gene, is one of the glycosyltransferases responsible for the presence of histo-blood group antigens on multiple organs and on the gastrointestinal mucosa.^11^ In recent years, genome-wide association studies have underlined the importance of FUT2 biology and showed that different polymorphisms may result in distinct secretor status, associated with the development of pathophysiology such as intestinal inflammation.^12^ Furthermore, FUT2 has been shown to have significant effects on the intestinal bacterial community composition.^12–15^ One of the major prototypical secretor-type oligosaccharides is the human milk oligosaccharide (HMO) 2’-fucosyllactose (2’FL).^16 17^ In vivo and in vitro studies showed that 2’FL exerts biological properties as prebiotic, antibacterial, antiviral and immunomodulating effects and modifies the host’s epithelial cell-surface glycome.^18^ This has prompted an increased interest in 2’FL as HMO source in infant formula and, more recently, 2’FL is also being investigated in pathological contexts.^19^ For example, in mice fed HFD, it was observed that 2’FL reduced body weight and fat mass gain.^20 21^ In addition, 2’FL protected against gut barrier disruptions induced by inflammatory stimuli, by increasing GCs number and *Muc2* expression.^22 23^ Further in vivo studies exploring the role of 2’FL on the mucus layer in the context of obesity induced by HFD feeding are still lacking.

To fill this gap, we designed a study aimed at deciphering whether the impact of 2’FL on metabolism could be linked to changes in the intestinal mucus production, glycosylation, secretion and degradation. In addition, we explored whether the effects on mucus layer and metabolism might be associated with modifications in gut microbiota composition, faecal proteome and endocannabinoid (eCB) system.

We believe that a comprehensive investigation into the intricate mechanisms of the mucus layer, including its biosynthesis, turnover and degradation, may offer novel insights into developing efficacious interventions for mitigating or preventing obesity and related metabolic disorders.

## Results

### 2’FL counteracts metabolic alterations induced by HFD

**Figure 1:**
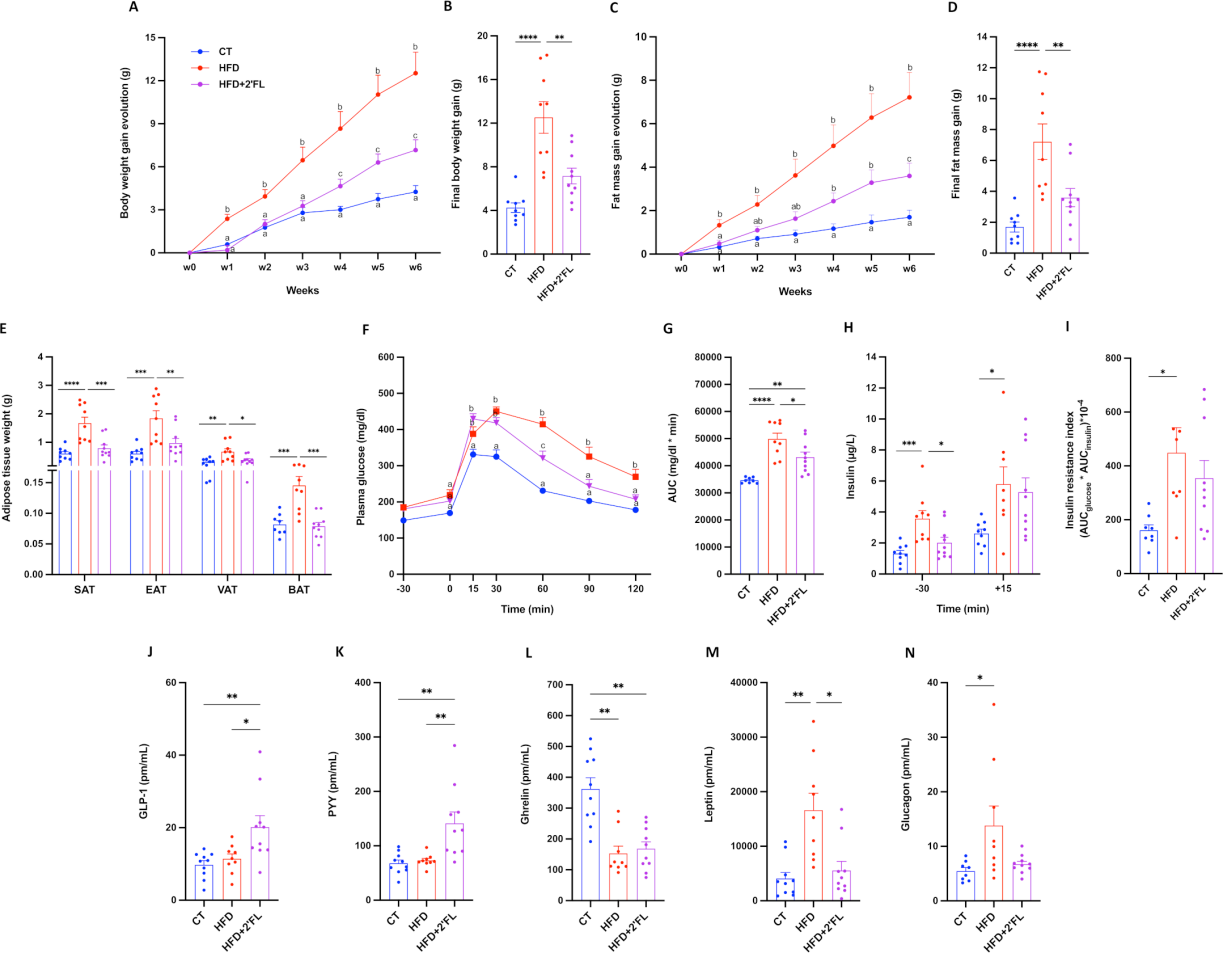
2’FL supplementation counteracts diet-induced obesity and glucose intolerance. (A) Body weight gain evolution and (C) fat mass gain evolution. (B) Final body weight gain and (D) fat mass gain. (E) Adipose tissue weights of subcutaneous (SAT), epidydimal (EAT), visceral (VAT) and brown (BAT) adipose tissue. (F) Plasma glucose (mg/dL) profile before and after 2 g/kg of glucose oral challenge measured during the oral glucose tolerance test (OGTT) and (G) the mean area under the curve (AUC) (mg/dL×min). (H) Plasma insulin (µg/L) measured 30 min before and 15 min after the glucose administration during the OGTT. (I) Insulin resistance index determined by multiplying the area under the curve (from −30 to 15 min) of blood glucose and plasma insulin obtained during the OGTT. (J–N) Plasma levels from the portal vein of glucagon-like peptide-1 (GLP-1), peptide YY (PYY), ghrelin, leptin and glucagon. Data are means±SEM (n=7–10/group). One-way ANOVA followed by Tukey post hoc test was applied to figure B, D, E, G–K, N while Kruskal-Wallis followed by Dunn’s test was applied to figure L,M, based on data distribution. Two-way ANOVA followed by Tukey post hoc test was applied to figure A, C, F. Data with different subscript letters are significantly different (p<0.05). *p<0.05; **p<0.01; ***p<0.001; ****p<0.0001. 2’FL, 2’-fucosyllactose; ANOVA, analysis of variance; HFD, high-fat diet.

Mice fed an HFD diet supplemented with 2’FL showed significantly lower body weight and fat mass gain (subcutaneous, epididymal, visceral and brown adipose tissues) compared with mice fed HFD alone (figure 1A–E). This could not be explained by food intake or lean/muscle mass since there were no differences between HFD and HFD+2’FL groups (online supplemental figure 1A-C). Additionally, 2’FL supplementation reduced glucose intolerance, as evidenced by the shape of the glycaemia curve during the oral glucose tolerance test and by the lower insulin levels in fasting state (figure 1F–I). These effects coincide with changes in hormones involved in metabolic pathways, since 2’FL significantly increased the concentration of glucagon-like peptide-1 (GLP-1) and peptide YY (PYY) and decreased leptin and glucagon (for the latter not significantly) while ghrelin was significantly reduced by the HFD, with no effects of 2’FL (figure 1J–N).

10.1136/gutjnl-2023-330301.supp2 Supplementary data

### 2’FL increases intestinal cells proliferation and markers involved in gut barrier function

**Figure 2:**
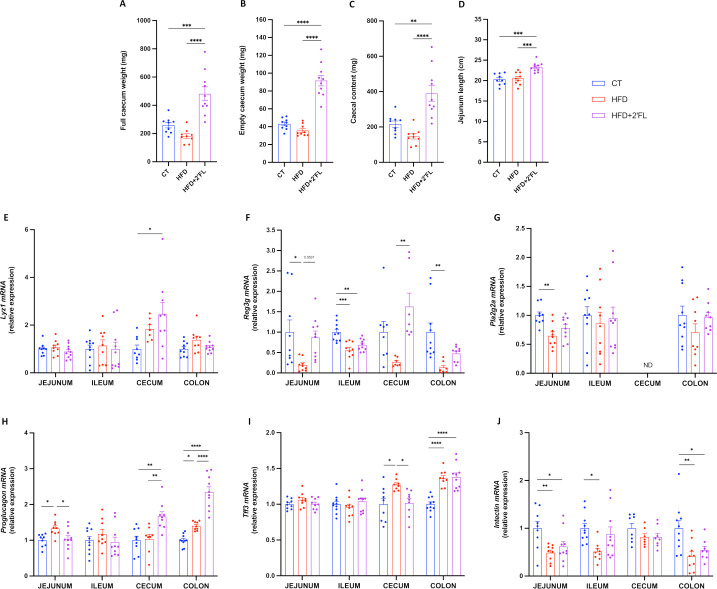
2’FL increases microbiota fermentation, intestinal cell proliferation and markers of the gut barrier. (A) Full caecum, (B) empty caecum and (C) caecal content weight. (D) Jejunum length. (E–J) mRNA relative expression of markers of the gut barrier function measured in the jejunum, ileum, caecum and colon. Antimicrobial peptides mRNA expression: (E) Lysozyme C (*Lyz1*), (F) Regenerating islet-derived 3-gamma (*Reg3g*), (G) Phospholipase A2 group II (*Pla2g2a*); (H) *Proglucagon*; (I) Trefoil factor 3 (*Tff3*); (J) *Intectin*. Data are means±SEM (n=7–12/group). Data were analysed using one-way ANOVA followed by Tukey post hoc test. *p<0.05; **p<0.01; ***p<0.001; ****p<0.0001. 2’FL, 2’-fucosyllactose; ANOVA, analysis of variance; ND, not detectable.

2’FL supplementation significantly increased full caecum and its content weight by about 80% and 150% compared with control and HFD, respectively (figure 2A–C). 2’FL supplementation also increased the length of the jejunum by almost 15% compared with CT and HFD (figure 2D).

Analysing the expression of genes involved in gut barrier function by qPCR, we found that 2’FL significantly increased the antimicrobial peptides *Lyz1* and *Reg3g* in the caecum, and *proglucagon* in the caecum and colon while it induced the expression of *Reg3g* in the jejunum and colon, *Pla2g2a* in the colon and *intectin* in the ileum, without reaching significance (figure 2E–J).

### 2’FL affects GCs differentiation and mucus production and secretion

**Figure 3:**
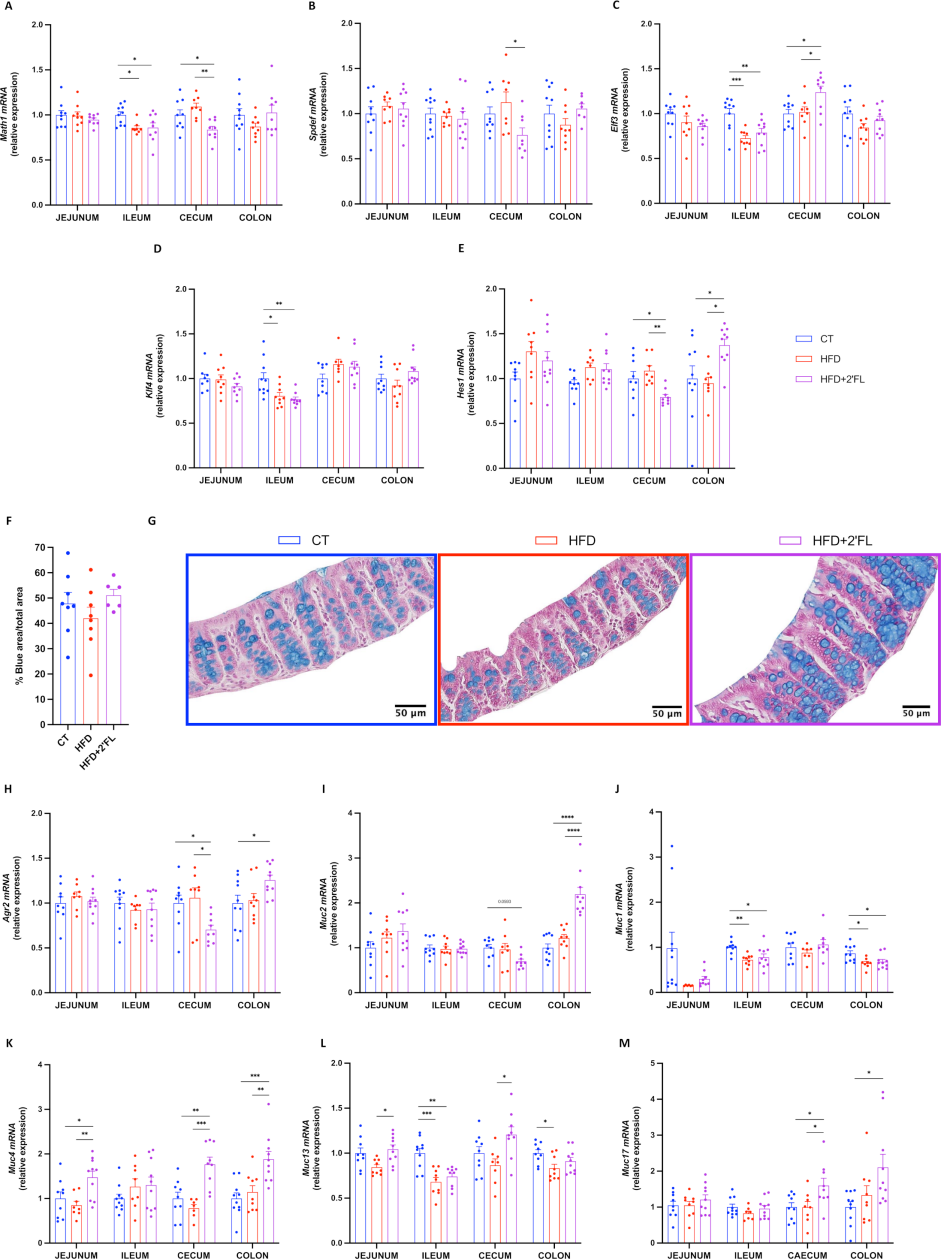
2’FL supplementation impacts on goblet cells and mucins production. (A–E) mRNA relative expression of transcriptional factors involved in the goblet cells differentiation, in the jejunum, ileum, caecum and colon: (A) atonal bHLH transcription factor 1 (*Math1*), (B) SAM pointed domain containing ETS transcription factor (*Spdef*), (C) E74 like ETS transcription factor 3 (*Elf3*), (D) kruppel like factor 4 (*Klf4*), hes family basic helix-loop-helix (bHLH) transcription factor 1 (*Hes1*). (F) Percentage of blue area on the total mucosal area in the proximal colon and (G) representative images for each group. (H–M) mRNA relative expression of markers involved in mucin production, in the jejunum, ileum, caecum and colon: (H) anterior gradient 2 (*Agr2*), (I) mucin 2 (*Muc2*), (J–M) mucin 1/4/13/17 (*Muc1*, *Muc4*, *Muc13, Muc17*). Data are means±SEM (n=6–12/group). One-way ANOVA followed by Tukey post hoc test or Kruskal-Wallis followed by Dunn’s test were applied based on data distribution. *p<0.05; **p<0.01; ***p<0.001; ****p<0.0001. 2’FL, 2’-fucosyllactose; ANOVA, analysis of variance; HFD, high-fat diet.

We next determined whether the effects of 2’FL supplementation on metabolism and gut barrier function were linked to changes in intestinal mucus. We showed that 2’FL significantly affected the expression of genes involved in GCs differentiation at different sites, with increased expression of *Elf3* in caecum and *Hes1* in colon, and decreased *Math1* and *Spdef* in caecum (figure 3A–E). In order to determine if the mucus inside the GCs was affected by the dietary treatments, we measured the proportion of the blue area (representing the mucins) over the total mucosal area, in histological sections using an Alcian blue staining. We found 22% more blue area in HFD+2’FL compared with HFD, though this difference did not reach significance (figure 3F,G).

Next, we set out to assess whether 2’FL treatment impacts intestinal mucins. We found that 2’FL significantly affected *Agr2* expression, required for the post-transcriptional synthesis and secretion of Muc2, which was decreased in caecum and increased in colon. In accordance with this observation, we also found a significant increase in *Muc2* expression (figure 3H,I). With regard to transmembrane mucins, 2’FL supplementation led to increased *Muc4* in jejunum, caecum and colon, *Muc13* in jejunum and caecum, and *Muc17* in caecum and colon (figure 3J–M). Furthermore, *Muc1* and *Muc13* expressions in the colon were negatively correlated with body weight and fat mass gain (online supplemental figure 2A,B).

**Figure 4:**
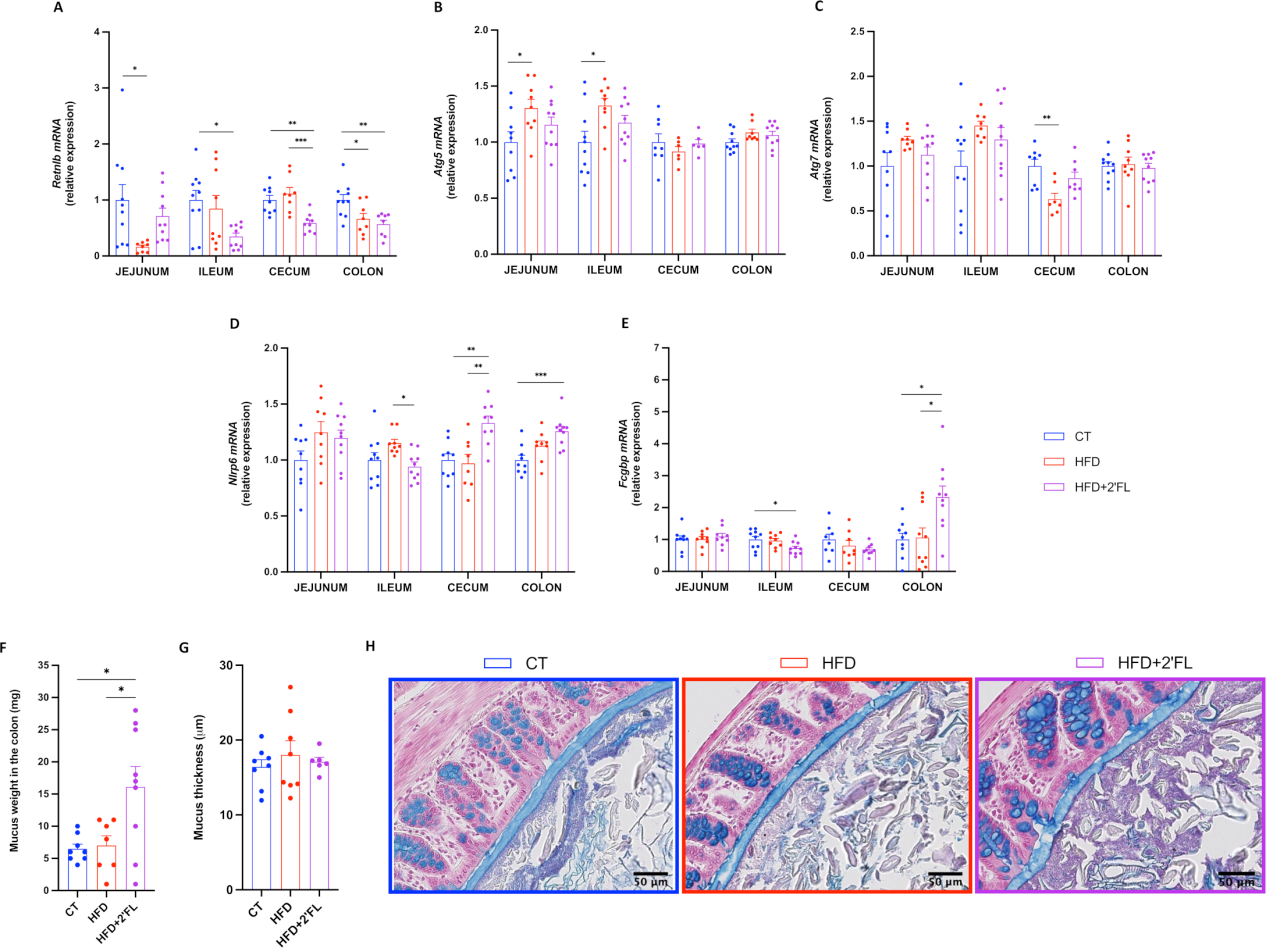
2’FL increases markers of mucus secretion. (A–E) mRNA relative expression of markers involved in the secretion of the mucus layer: (A) resistin-like beta (*Retnlb*), (B) autophagy protein 5 (*Atg5*), (C) autophagy protein 7 (*Atg7*), (D) NOD‐like receptor family pyrin domain containing 6 (*Nlrp6*), (E) Fc gamma binding protein (*Fcgbp*). (F) Weight of the mucus in the colon after scraping in milligrams. (G) Mucus thickness in the proximal colon measured by ImageJ (in micrometre) and (H) representative images for each group. Data are means±SEM (n=6–12/group). One-way ANOVA followed by Tukey post hoc test or Kruskal-Wallis followed by Dunn’s test were applied based on data distribution. *p<0.05, **p<0.01, ***p<0.001. 2’FL, 2’-fucosyllactose; ANOVA, analysis of variance; HFD, high-fat diet.

Finally, we observed that dietary treatments differentially affected the expression of genes involved in intestinal mucus secretion and stabilisation. In particular, 2’FL supplementation tended to increase *Retnlb* in jejunum but decreased in the other intestinal segments. 2’FL supplementation increased two other key markers, *Nlrp6* in caecum and colon and *Fcgbp* in colon while slightly counteracting the effects of the HFD on the expression of *Atg5* and *Atg7* (figure 4A–E).

Although there was no difference in mucus thickness as assessed on histological sections (figure 4G,H), we found a significant higher weight of the mucus collected by scraping the colon in mice supplemented with 2’FL, suggesting a potential increase in mucus production (figure 4F).

**Figure 5:**
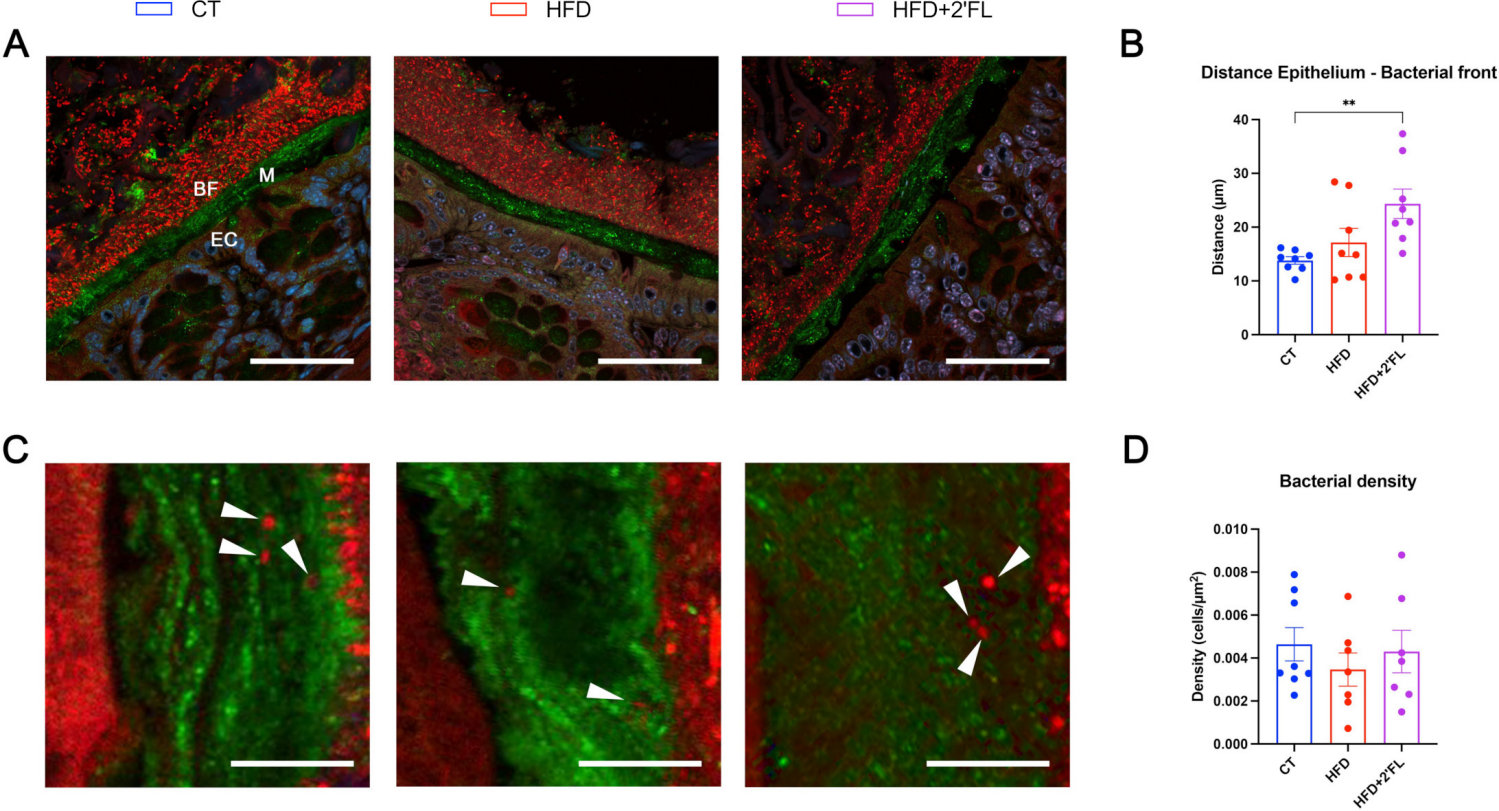
Pictures representative of the bacterial penetration assessed by measuring the distance between the bacterial front and the epithelial cells (A, B) and the bacterial density in the inner mucus layer (C, D). (A) Mouse distal colon section in which Muc2C3 immunostaining shows the Muc2-positive mucus layer on the epithelium. The inner mucus layer (M) is almost completely devoid of bacteria, which are visualised by a FISH approach using a general bacterial probe conjugated with C3 (red), whereas the outer mucus layer contains large concentrations of bacteria with a clearly delineated bacterial front (BF). The sections are counterstained with DAPI to visualise nuclei (blue). Epithelial cells (EC) emit some autofluorescence making them visible (Scale bar: 50 µm). (B) Quantitative measurement of the spatial separation between the epithelial cells and the bacterial front. (C) Magnification (×20) of the inner mucus layer and of penetrating bacteria. Epithelial cells are on the left, while the bacterial front is on the right (scale bar: 10 µm). (D) Quantification of the bacterial density in the inner mucus layer (number of bacterial cells counted divided by the surface area of mucus). Data are means±SEM (n=7–8/group). Arrow heads show bacteria in red. Data were analysed using Kruskal-Wallis test followed by Dunn’s test. **p<0.01. FISH, fluorescence in situ hybridisation; HFD, high-fat diet.

Using a fluorescence in situ hybridisation (FISH) approach against 16S RNA to detect bacteria combined with a Muc2C3-specific staining of the mucus on colon sections, we observed an abrupt change from the inner to outer mucus layer with bacterial concentrations jumping from almost virtually free of bacteria to a high density without any perceptible gradient. The bacterial front was found to be morphologically intact in all groups. The thickness of the bacteria-free mucus was not statistically different between CT and HFD groups, though we observed a significant increase in the 2’FL treated group compared with the control group (p=0,01 Kruskal-Wallis test) (figure 5A).

When focusing on the apparent virtually free of bacteria inner mucus layer, we found that some bacteria, though very few, were able to penetrate it. We quantified the density by counting these cells and normalising to the area of mucus, but we found no differences between groups (figure 5B).

### 2’FL affects mucin glycan profile

**Figure 6:**
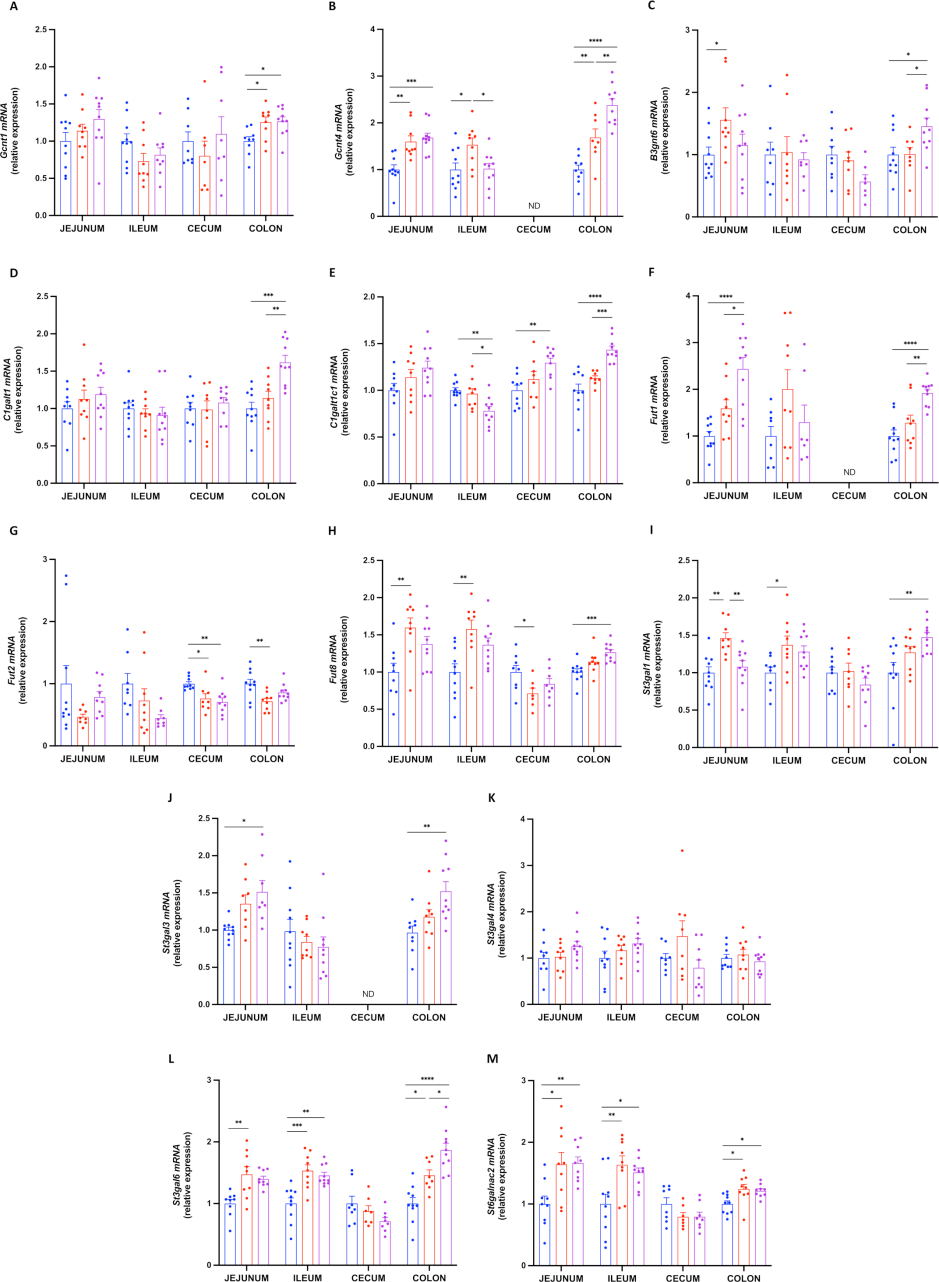
2’FL increases the expression of glycosyltransferases involved in mucin glycosylation. mRNA relative expression of glycosyltransferases in the jejunum, ileum, caecum and colon: (A) glucosaminyl (*N*-acetyl) transferase 1 (*Gcnt1*), (B) glucosaminyl (*N*-acetyl) transferase 4 (*Gcnt4*), (C) UDP-GlcNAc:betaGal beta-1,3-*N*-acetylglucosaminyltransferase 6 (*B3gnt6*), (D) core one synthase, glycoprotein-*N*-acetylgalactosamine 3-beta-galactosyltransferase 1 (*C1galt1*), (E) C1GALT1 specific chaperone 1 (*C1galt1c1*), (F–H) fucosyltransferase 1/2/8 (*Fut1*, *Fut2*, *Fut8*), (I–M) ST3 b-galactoside a-2,3-sialyltransferase 1/3/4/6 (*St3gal1*, *St3gal3*, *St4gal4*, *St3gal6*), (O) ST6 *N*-acetylgalactosaminide a-2,6-sialyltransferase 2 (*St6galnac2*). Data are means±SEM. (n=7–12/group). Data were analysed using one-way ANOVA followed by Tukey post hoc test. *p<0.05; **p<0.01; ***p<0.001; ****p<0.0001. 2’FL, 2’-fucosyllactose; ANOVA, analysis of variance; ND, not detectable.

**Figure 7:**
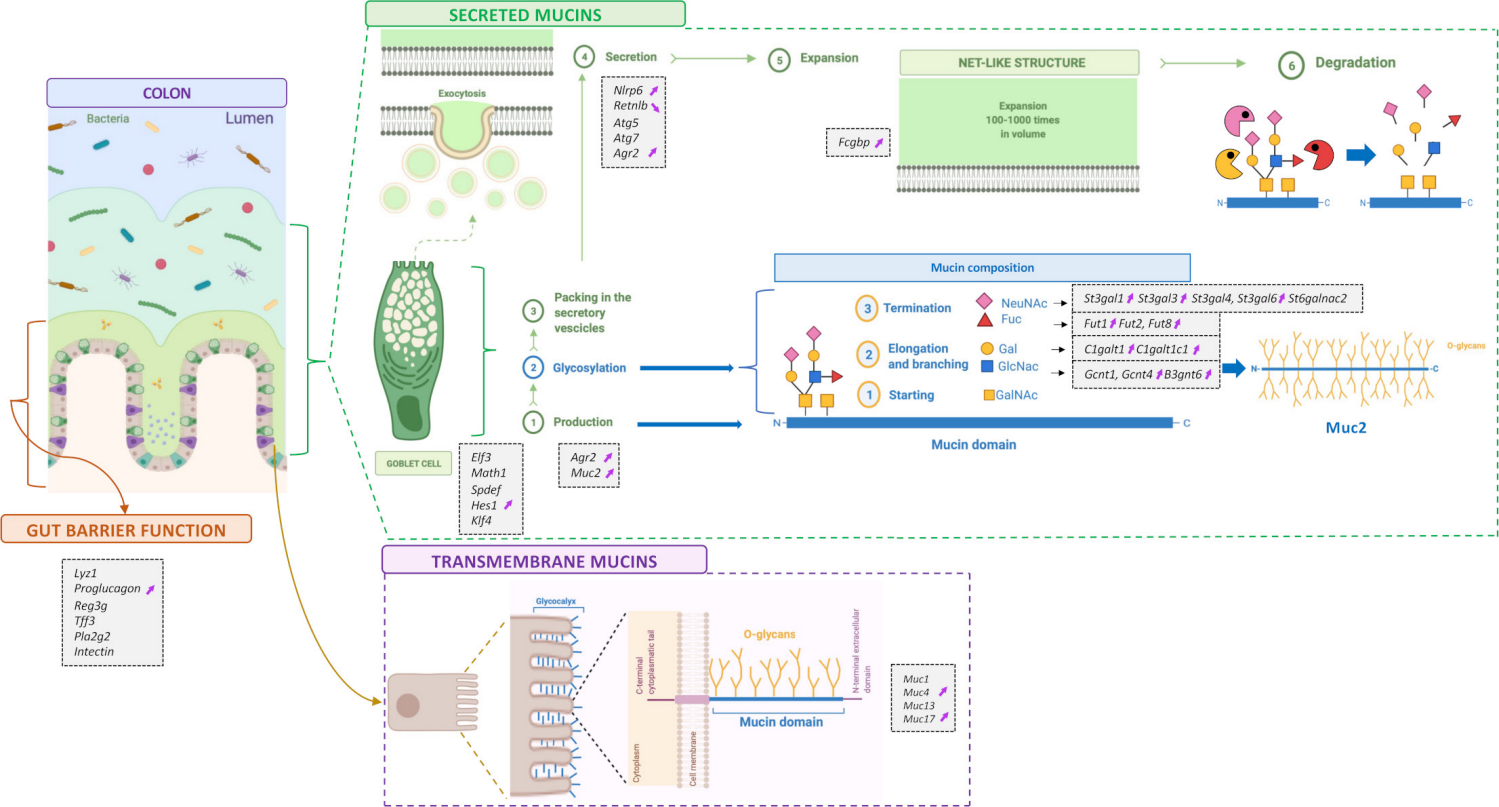
Schematic figure summarising the expression analysis of 35 genes in the jejunum, ileum, caecum and colon. Markers involved in gut barrier function and mucins production, glycosylation and secretion measured by RT-qPCR. Markers are enclosed in small grey boxes. Purple arrows indicate those that significantly changed due to 2’FL supplementation in the colon. 2’FL, 2’-fucosyllactose.

To determine whether HFD and 2’FL supplementation affected mucin glycosylation, we first measured the expression of glycosyltransferases involved in elongation, branching and termination of the mucin glycan chain. We found that 2’FL significantly increased *Gcnt4*, *B3gnt6* and *C1galt1* in colon, *C1galt1c1* in caecum and colon, *Fut1* in jejunum and colon, *Fut8* and *St3gal1* in colon, *St3gal3* in jejunum and colon and *St3gal6* in colon (figure 6A–M). Interestingly, *Fut2* was decreased by the HFD in caecum and colon, but not affected by 2’FL supplementation. All the data from the mRNA expression described in the colon are schematised in figure 7.

**Figure 8:**
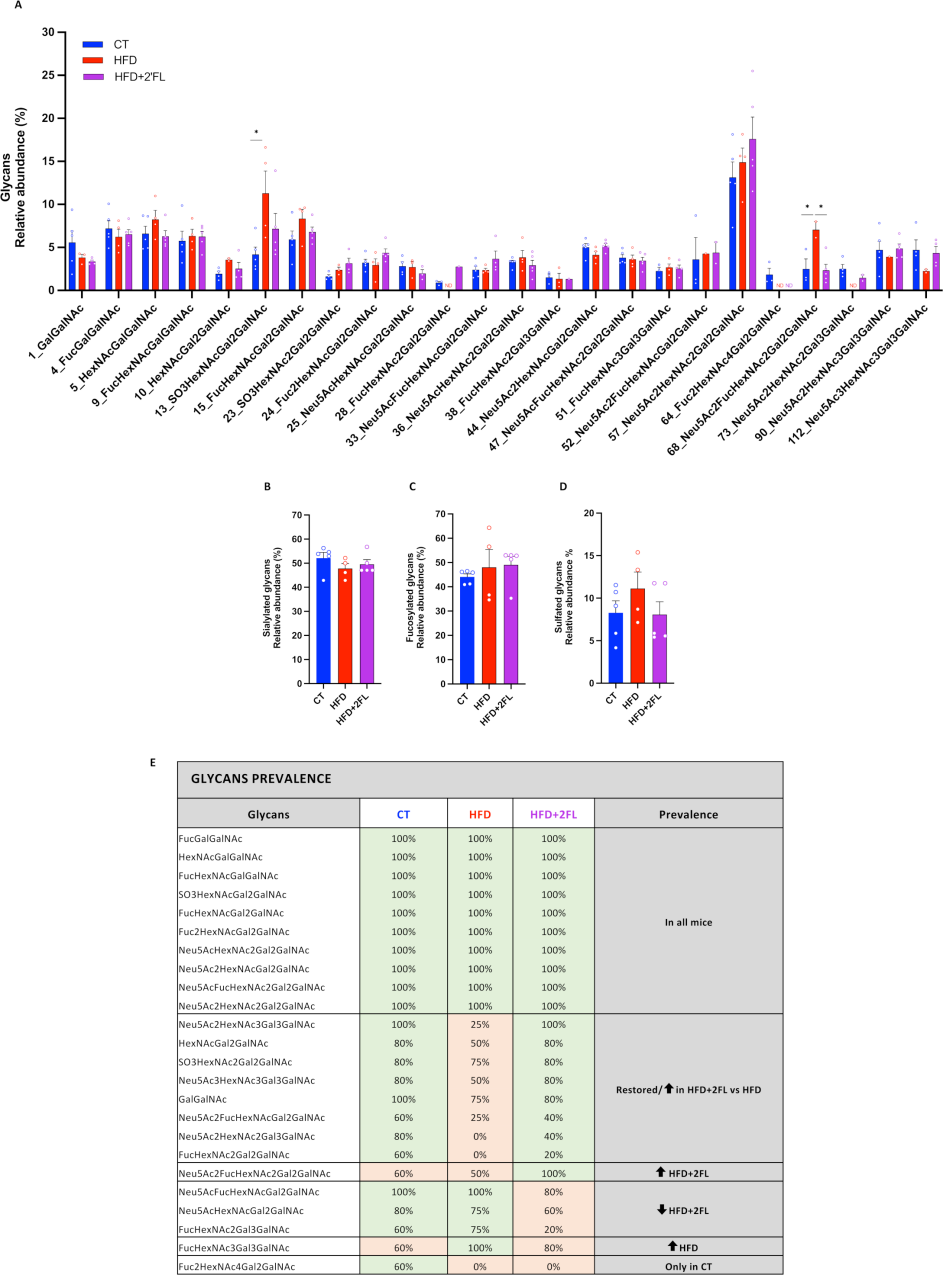
High-fat diet and 2’FL supplementation affects mucin glycans composition in the colon. (A) Glycan relative abundance in percentage; relative abundance of (B) sialylated glycans, (C) fucosylated glycans and (D) sulfated glycans. (E) Glycan prevalence calculated by dividing the number of mice for which the glycan was present for the total number of mice in the group. Only glycans present in at least 3 mice and in at least one group are shown. Data are means±SEM (n=4–5/group). Data were analysed using Kruskal-Wallis followed by Dunn’s test. *p<0.05. 2’FL, 2’-fucosyllactose; HFD, high-fat diet; ND, not detectable.

We next analysed mucin glycosylation by tandem mass spectrometry (MS/MS) and found that two of them were significantly higher in HFD, compared with the CT and/or HFD+2’FL group (figure 8A–D). Figure 8E shows that 10 glycans were present in all the mice, 8 had a lower prevalence in the HFD group only or were restored following supplementation with 2’FL, and 3 were less prevalent in the HFD+2’FL group.

### 2’FL affects the endocannabinoid system

**Figure 9:**
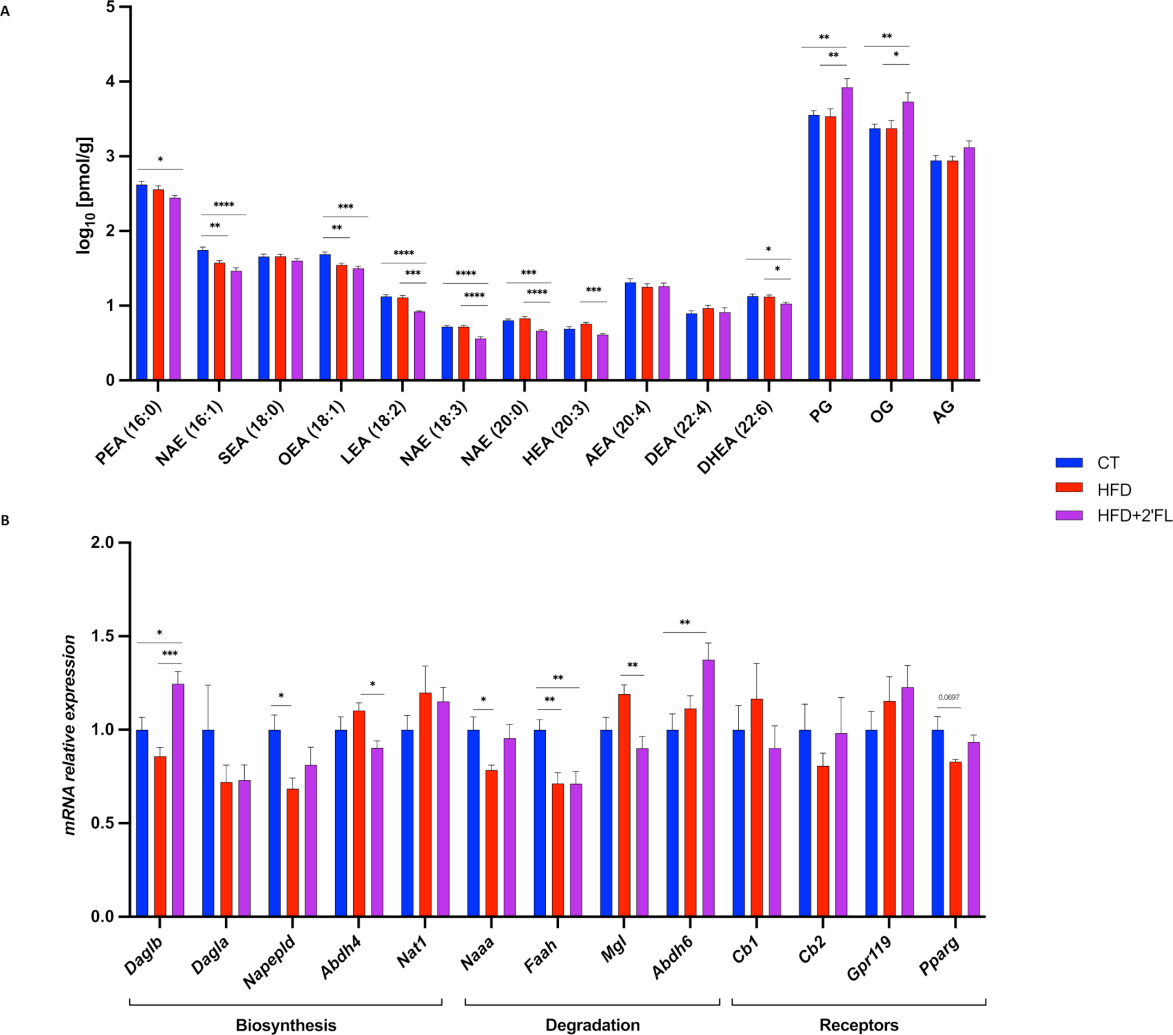
Different caecal eCBome tone in 2’FL supplemented mice. (A) Concentrations of the eCBome-related mediators in the caecal tissue (pmol/g wet tissue weight) measured by ultra-high-performance liquid chromatography–tandem mass spectrometry (UHPLC-MS/MS). (B) mRNA relative expression of receptors and metabolic enzymes for monoacylglycerols and *N*-acylethanolamines measured by RT-qPCR. Data are means±SEM (n=9–10/group). Data were analysed using one-way ANOVA followed by Tukey post hoc test. *p<0.05; **p<0.01; ***p<0.001; ****p<0.0001. 2’FL, 2’-fucosyllactose; Abdh4, alpha/beta-hydrolase 4; Abdh6, α/β-Hydrolase domain-containing 6; AEA, *N*-arachidonoylethanolamine; AG, 2-arachidonoylglycerol; ANOVA, analysis of variance; Cb1/Cb2, cannabinoid type 1/2 receptors; DEA, *N*-docosatetraenylethanolamine; Dagla, diacylglycerol lipase-alpha; Daglb, diacylglycerol lipase beta; DHEA, *N*-docosahexaenoylethanolamine; Faah, fatty-acid amide hydrolase; Gpr119, G-protein-coupled receptor 119; HEA, *N*-homo-linolenylethanolamine; LEA, *N*-linoleylethanolamine; Mgl, monoacylglycerol lipase; Naaa, *N*-acylethanolamine acid amidase; NAE, *N*-acylethanolamine; Napepld, N-acyl phosphatidylethanolamine phospholipase D; Nat1, N-acetyltransferase 1; OEA, *N-*oleoylethanolamine; OG, mono-oleoylglycerol; PEA, *N*-palmitoylethanolamine; PG, mono-palmitoylglycerol; Pparg, peroxisome proliferator-activated receptor gamma SEA, *N*-stearoylethanolamine.

We previously discovered that different bioactive lipids belonging to the eCB system are able to exert control over the gut microbiota and the gut barrier function.^2 24–27^ Hence, we measured caecal levels of eCBs (arachidonoylglycerol (AG) and anandamide (NAE 20:4)) and related *N*-acylethanolamines, and found that HFD+2’FL mice had significant lower levels of NAEs (16:1, 18:3, 20:0), LEA, OEA, PEA, DHEA and HEA, compared with CT and/or HFD mice. While, they had significant higher levels of mono-oleoylglycerol (OG) and mono-palmitoylglycerol (PG) (figure 9A). 2’FL affected the expression of genes involved in the biosynthesis and degradation of eCBs, by significantly upregulating *Daglb* and *Abdh6*, and downregulating *Abdh4*, *Faah* and *Mgl* (figure 9B).

### 2’FL changes gut microbiota composition

**Figure 10:**
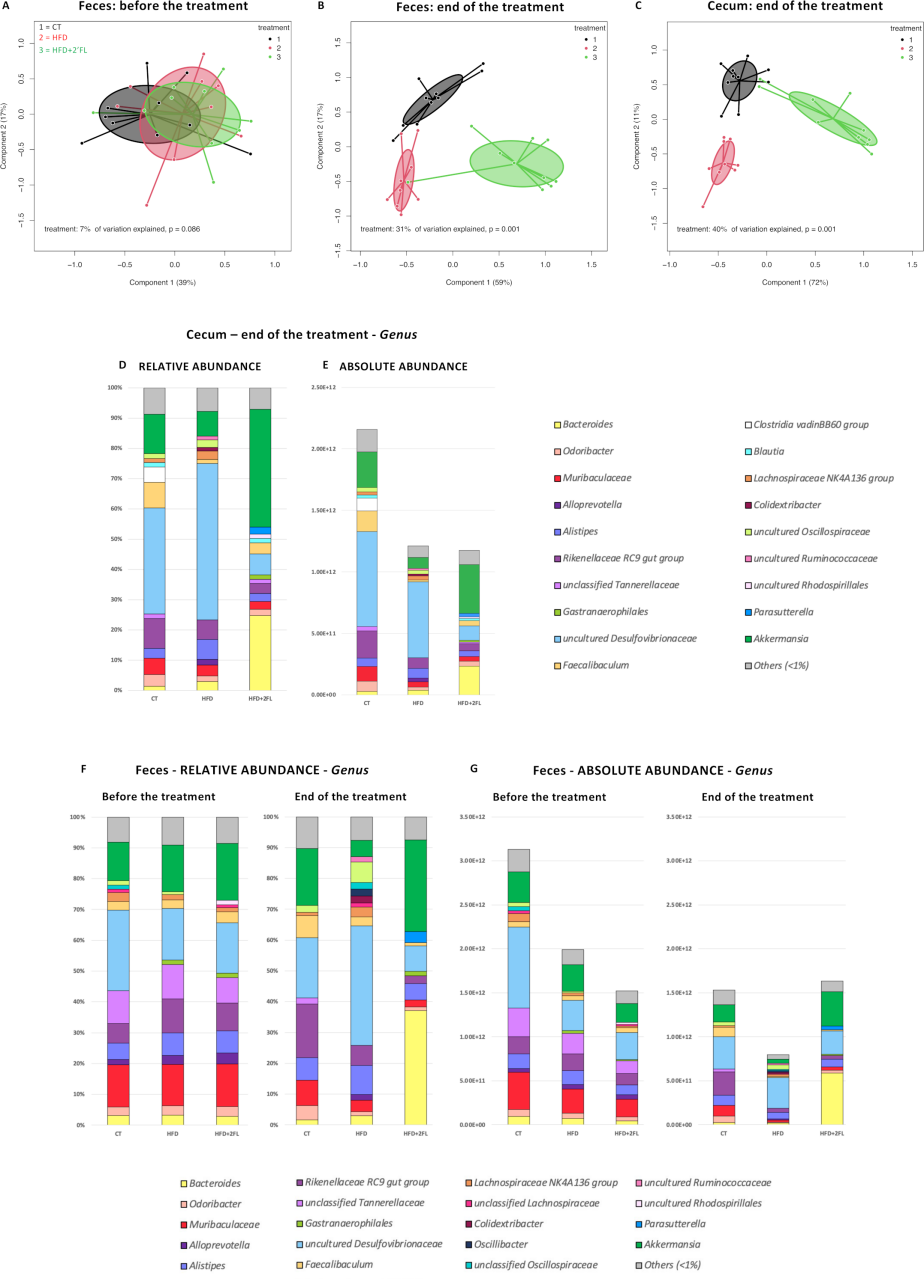
2’FL induces changes in the caecal and faecal gut microbiota composition. Principal coordinates analysis (PCoA) plot of the gut microbiota, in which mice are grouped by treatment, based on the Bray-Curtis dissimilarity in (A) faeces before the treatment (B) faeces at the end of the treatment and (C) caecum at the end of the treatment (n=9–10/group). (D–G) Bar graphs showing grouped taxonomic profiles of the gut bacteria at the genus level: (D, E) relative and absolute abundance in the caecum, at the end of the treatment; (F, G) relative and absolute abundance in the faeces, before and at the end of the treatment (n=9–10/group). Only the bacterial genera with >1% relative abundance are shown; the rest are indicated as ‘others (<1%)’. 2’FL, 2’-fucosyllactose; HFD, high-fat diet.

Before the treatment all mice shared a similar faecal microbiota composition, while in the end both the faecal and caecal microbiota profiles were significantly clustered based on the diets (figure 10A–C). The results shown below refer to changes observed in both relative and absolute abundance.

At the phylum level, the caecal gut microbiota of CT and HFD groups was dominated by Desulfobacterota while HFD+2’FL by Bacteroidota and Verrucomicrobiota. In the faeces, the CT group was dominated by Bacteroidota, HFD by Desulfobacterota and HFD+2’FL by Bacteroidota and Verrucomicrobiota (online supplemental figure 3A–D and online supplemental tables 1 and 2).

10.1136/gutjnl-2023-330301.supp3 Supplementary data

10.1136/gutjnl-2023-330301.supp4 Supplementary data

At the genus level, the caecal gut microbiota was enriched in uncultured *Desulfovibrionaceae* in the CT and HFD groups (35.1 and 51.7%, respectively), whereas *Akkermansia* and *Bacteroides* were the dominant genera in the HFD+2’FL group (39% and 24.8%, respectively) (figure 10D,E). Similarly, the faecal gut microbiota was dominated by uncultured *Desulfovibrionaceae*, *Rikenellaceae* RC9 gut group and *Akkermansia* in the CT group (19.4%, 17.3% and 18.4%, respectively), by uncultured *Desulfovibrionaceae* in the HFD group (38.8%), and *Bacteroides* and *Akkermansia* in the HFD+2’FL group (37.1% and 29.8%, respectively) (figure 10F,G) .

**Figure 11:**
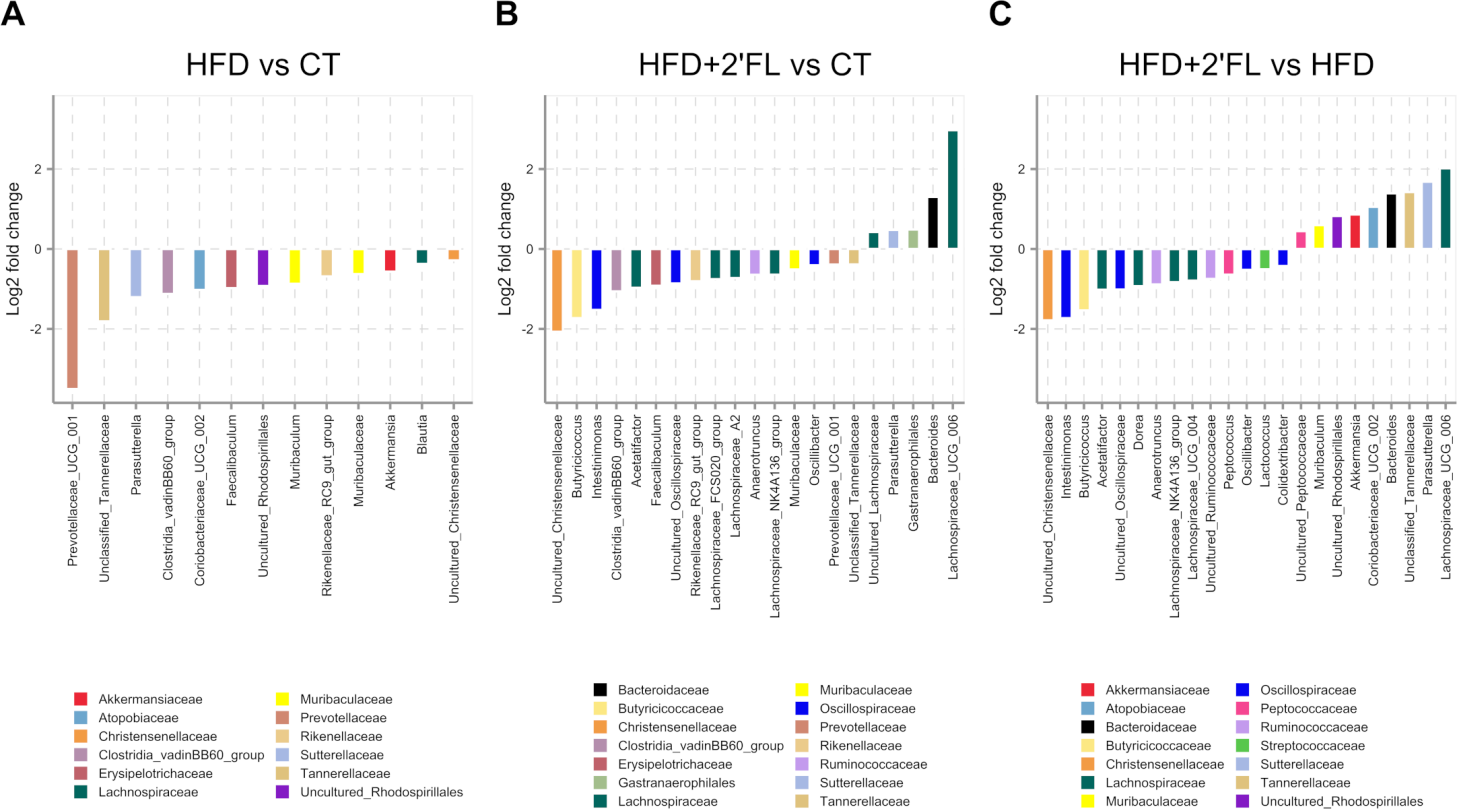
Bacterial genera significantly differed in absolute abundance (FDR-corrected p<0.05) in (A) HFD compared with CT (log2 fold change values calculated relative to CT), (B) HFD+2’FL compared with CT (log2 fold change values calculated relative to CT) and (C) HFD+2’FL compared with HFD (log2 fold change values calculated relative to HFD). Bar colour and bottom legend denote family-level taxonomic classification. See online supplemental table 3 for full results. 2’FL, 2’-fucosyllactose; HFD, high-fat diet.

Notably, HFD-fed mice had significant lower levels of *Akkermansia*, *Parasutterella*, unclassified *Tannerellaceae*, *Muribaculaceae* and *Rikenellaceae* RC9 gut group compared with CT mice while 2’FL treatment significantly increased *Akkermansia*, *Parasutterella*, unclassified *Tannerellaceae* and *Bacteroides* compared with HFD only, in the faeces (figure 11A–C, online supplemental table 3).

10.1136/gutjnl-2023-330301.supp5 Supplementary data

### 2’FL affects bacterial glycosidases and faecal proteome

**Figure 12:**
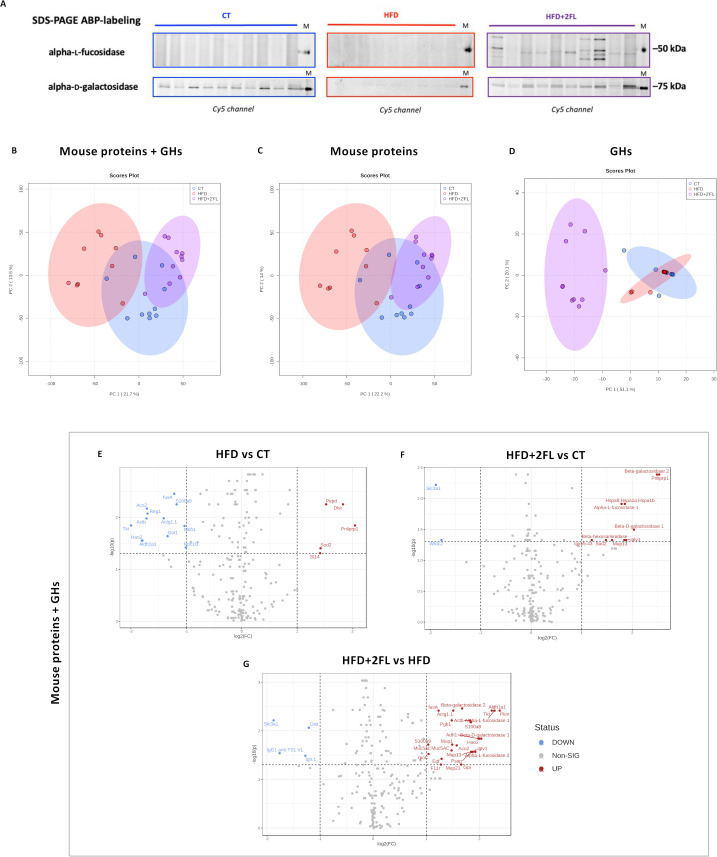
High-fat diet and 2’FL supplementation affects faecal proteome. (A) Cy5-ABP-labelling of alpha-L-fucosidase and alpha-D-galactosidase from mouse faecal extract (1 µg of proteins; 1 µM α-L-fucosidase and 0.5 µM α-galactosidase). Principal component analysis (PCA) of (B) faecal mouse proteins and GHs together, of (C) mouse proteins only and of (D) GHs separately. (E–G) Volcano plot comparing the different groups together, including mouse proteins and GHs. PCA and volcano plot were done with MetaboAnalyst (n=9–10/group). 2’FL, 2’-fucosyllactose; GHs, glycosyl hydrolases; HFD, high-fat diet.

To evaluate the mucus degradation by the gut microbiota, we investigated bacterial GHs alpha-L-fucosidase and alpha-D-galactosidase by in-gel fluorescent activity-based probes (ABP) labelling.^28 29^ We found ABP-labelling for alpha-L-fucosidase only in the HFD+2’FL group, with mice within this group displaying different profiles. While, alpha-D-galactosidase labelling was present in CT and HFD+2’FL, without any signals in HFD (figure 12A).

To further confirm the presence of GHs, we analysed the total faecal proteome, using a bespoke database containing mouse proteins and GHs involved in mucin glycan degradation: fucosidases, galactosidases, hexosaminidases and sialidases. The principal component analysis (PCA) showed different clustering between HFD and HFD+2’FL (figure 12B–D). Particularly, when taking only GHs into account, CT and HFD displayed overlapping clusters, while HFD+2’FL cluster was completely separated. The volcano plot showed that HFD feeding significantly changed the abundance of 17 proteins, while 2’FL supplementation changed 12 proteins compared with CT and 30 compared with HFD (figure 12E–G).

**Figure 13:**
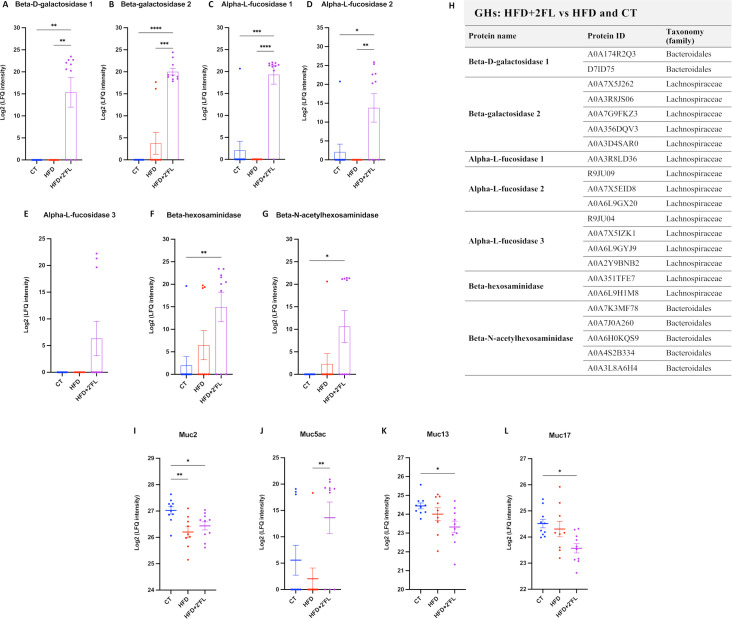
2’FL supplementation affects faecal bacterial GHs and mucins. Bacterial GHs and (H) their relative protein ID and taxonomy (family). (I–L) Mucins (Muc2, Muc5ac, Muc13, Muc17). One-way ANOVA followed by Tukey post hoc test or Kruskal-Wallis followed by Dunn’s test was applied based on data distribution. *p<0.05; **p<0.01; ***p<0.001; ****p<0.0001. 2’FL, 2’-fucosyllactose; ANOVA, analysis of variance; GHs, glycosyl hydrolases; HFD, high-fat diet; ND, not detectable.

Interestingly, 2’FL supplementation significantly upregulated beta-galactosidase, alpha-L-fucosidase, beta-hexosaminidase and beta-*N*-acetylhexosaminidase, belonging to *Bacteroidales* and *Lachnospiraceae* bacterial families (figure 13A–H).

In addition to changes in GHs, dietary treatments affected several faecal mucins. Indeed, Muc2 was significantly lower in HFD and HFD+2’FL groups compared with the CT group, and Muc13 and Muc17 were significantly lower in HFD+2’FL compared with CT; while Muc5ac was significantly higher in HFD+2’FL compared with HFD (figure 13I–L).

Taking into account the results from the Wilcoxon rank-sum test, we found that HFD significantly changed 78 proteins compared with CT, while supplementing 2’FL changed 90 proteins compared with HFD (online supplemental table 4).

10.1136/gutjnl-2023-330301.supp6 Supplementary data

**Figure 14:**
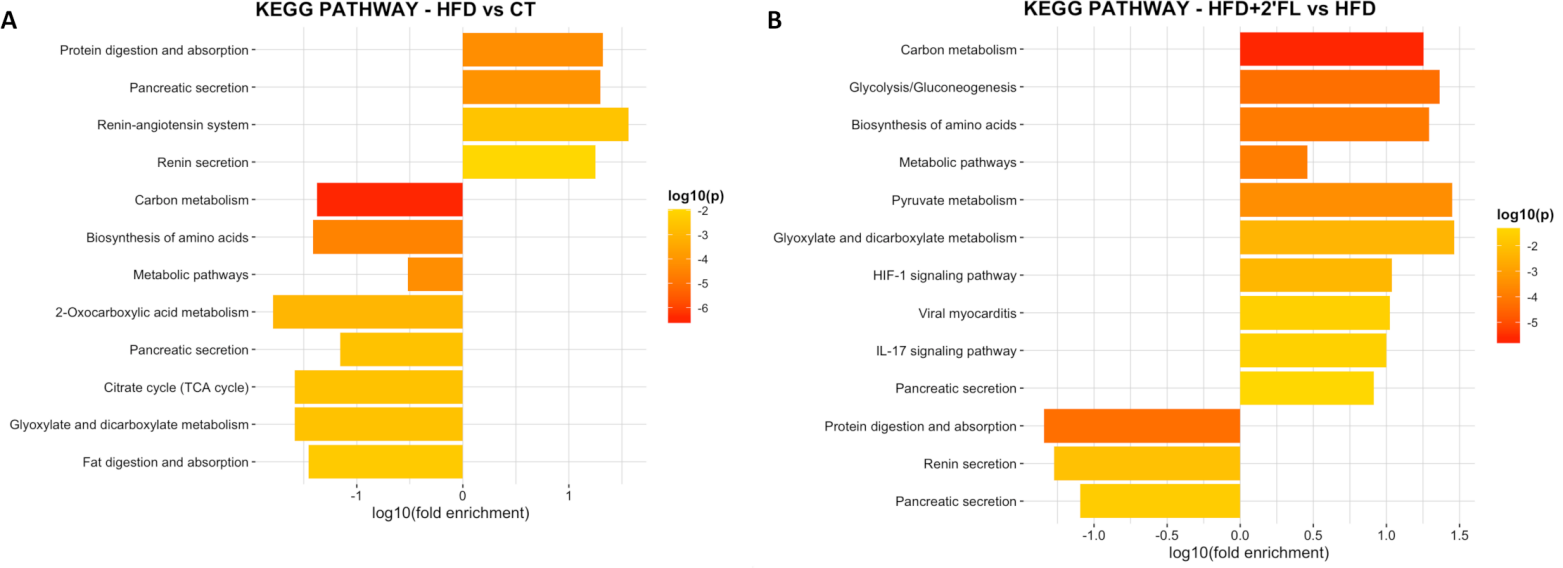
KEGG pathway enrichment analysis performed by using the Database for Annotation, Visualization and Integrated Discovery (DAVID) database. Only significant upregulated and downregulated terms (p<0.05) are shown. 2’FL, 2’-fucosyllactose; HFD, high-fat diet.

By executing KEGG pathway enrichment of mouse proteins, we observed that HFD feeding significantly upregulated proteins involved in protein digestion and absorption while it significantly downregulated proteins involved in carbon metabolism, biosynthesis of amino acids, metabolic pathways, fat digestion and absorption, and others (figure 14A). Notably, 2’FL supplementation reversed all the changes induced by HFD (figure 14B, online supplemental figure 4).

### Humans proteomic

**Figure 15:**
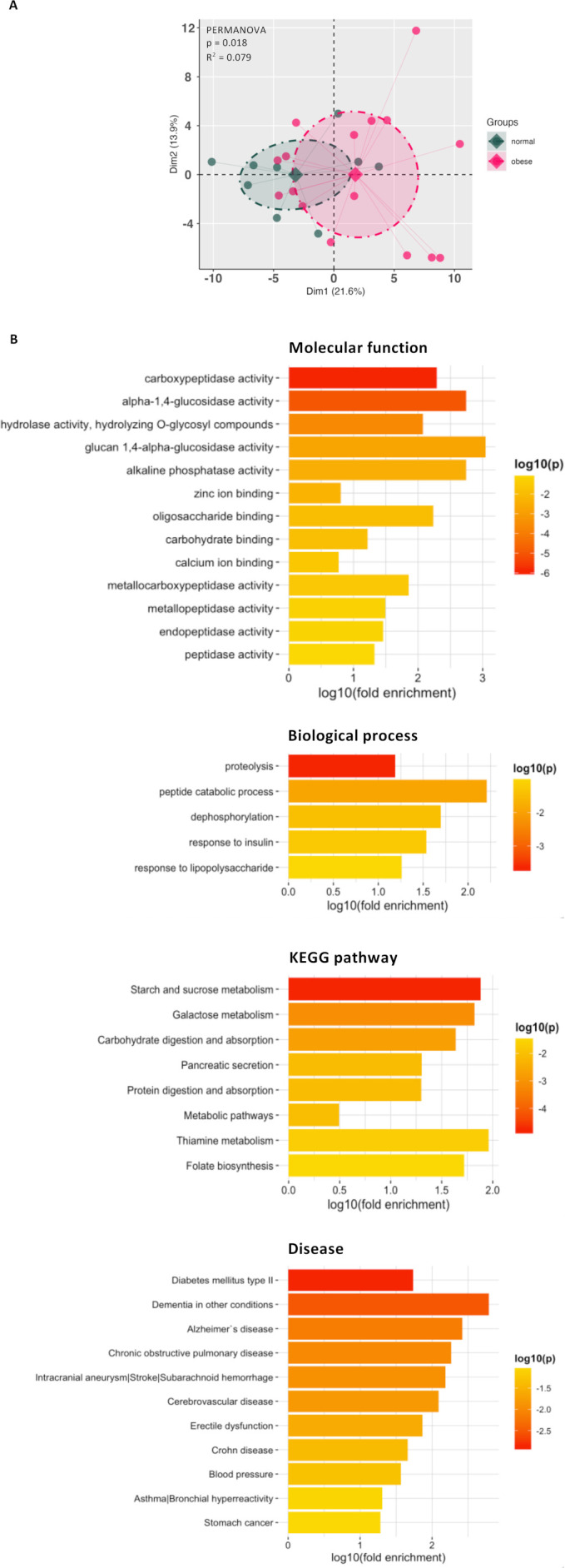
(A) Results of the principal component analysis and permutational multivariate analysis of variance (PERMANOVA) for normal (n=9) and obese (n=16) human subject’s proteomes. (B) Enrichment of molecular function, biological process, KEGG pathway and disease in terms of gene ontology (GO) categories. GO categories were determined using DAVID.

The results observed in rodents let us wondering if similar changes could be found in humans. We analysed the faecal proteome of lean and obese subjects and found that, among 133 proteins, 17 were significantly changed and increased in obese subjects (figure 15A; online supplemental tables 5 and 6). By doing functional annotation clustering, we observed that these proteins were linked to the enrichment of terms that were also enriched in HFD-fed mice (online supplemental figure 5, online supplemental table 7). Interestingly, by investigating the molecular function, biological process, KEGG pathway and disease, we found that they were involved in metabolic processes and diseases, such as type 2 diabetes (figure 15B, online supplemental table 8).

10.1136/gutjnl-2023-330301.supp7 Supplementary data

10.1136/gutjnl-2023-330301.supp8 Supplementary data

10.1136/gutjnl-2023-330301.supp9 Supplementary data

10.1136/gutjnl-2023-330301.supp10 Supplementary data

## Material and methods

See online supplemental materials and methods.

10.1136/gutjnl-2023-330301.supp1 Supplementary data

## Discussion

In this study, we found that 2’FL counteracted diet-induced obesity and metabolic alterations together with affecting mucus production, secretion and glycosylation, as well as gut microbiota composition, bacterial GHs, faecal proteome and eCB system.

Previous studies showed that 2’FL reduced energy intake, body weight or fat mass, in mice fed HFD, without affecting plasma glucose.^20 21^ Here, we found that HFD-fed mice supplemented with 2’FL had significantly lower body weight gain, fat mass gain, plasma glucose and insulin levels. These effects could partially be explained by a change in different hormones involved in appetite regulation and energy metabolism. Indeed, GLP-1 and PYY were significantly higher and leptin and glucagon were significantly lower in mice supplemented with 2’FL.

HFD feeding and obesity have been associated with gut barrier disruption, increased lipopolysaccharide translocation and metabolic endotoxaemia.^1^ Supplementing 2’FL to HFD has shown protective effects on markers of the gut barrier, but the mechanisms were not explored.^20 21^ In this study 2’FL supplementation led to higher expression of antimicrobial peptides *Lyz1* and *Reg3g*, and *proglucagon*, the precursor of GLP-1 and GLP-2, involved in improved gut barrier function, in specific sites of the gastrointestinal tract.

To further explore the mechanisms involved in gut barrier regulation, we focused on the mucus layer. In vitro studies reported that 2’FL led to enhanced *MUC2* expression and secretion on human GCs during inflammatory conditions.^23 30^ While, in vivo, 2′FL ameliorated colitis by recovering GC numbers and improving *Muc2* expression in mice.^22 23^ However, no studies investigated the effect of 2’FL supplementation on the intestinal mucus in the context of HFD feeding and obesity. Our data showed that 2’FL supplementation led to increased expression of several markers involved in GCs differentiation (eg, *Elf3* and *Hes1*) and synthesis and secretion of the main component of the mucus layer (ie, *Agr2*, *Muc2*). In addition, several markers related to mucus secretion and stabilisation were also increased (eg, *Retnlb*, *Nlrp6* and *Fcgbp*). These effects were linked to a higher quantity of mucus collected in the colon of mice receiving 2’FL, and with GCs more filled with mucus. The mucus penetrability to bacteria assessed by two parameters (ie, the distance from the bacterial front to the epithelial cells and the density of bacterial cells within the inner mucus layer) did not show significant differences between CT and HFD groups, though we observed an increased mucus layer thickness in the 2’FL treated group compared with the CT, suggesting that 2’FL protects the epithelium against bacteria penetration.

In addition to the secreted mucins, other important components of the gut barrier are transmembrane mucins. We found that 2’FL supplementation significantly upregulated the expression of *Muc4*, *Muc13* and *Muc17* in different intestinal compartments. Interestingly, *Muc1* and *Muc13* expressions in the colon were negatively correlated with body weight and fat mass gain (online supplemental figure 2A,B), suggesting their potential involvement in metabolic processes. However, their role in the context of obesity and metabolic disorders is still unknown, as only a few studies focused on these aspects. Two studies showed that 2′FL impacted glycocalyx average thickness and increased mRNA levels of *Muc1* in the presence of *Escherichia coli* challenge.^31 32^ To our knowledge, no other data on transmembrane mucins and 2’FL are available.

The composition of mucin glycans in the intestine has been shown to be important for microbial colonisation.^10^ Glycosyltransferases are the enzymes responsible for mucin glycosylation and it has been suggested that they are affected by dietary treatments, which could impact Muc2 glycosylation.^33^ Here, we found that 2’FL supplementation significantly affected the expression of glycosyltransferases, mainly in the colon where 9 out of 13 of them were upregulated. Such changes were also observed in a previous study following supplementation of mice with fructooligosaccharides.^34^ Interestingly, we found that *Fut2* expression in the colon negatively correlated with body weight gain and fat mass gain (online supplemental figure 2C). Together with previous studies showing that *Fut2* mutation led to liver disease, these findings suggest that it could probably be involved in metabolic processes.^35 36^

Based on these results, we asked whether mucin glycan composition could be affected by dietary intervention. Previous studies showed that HFD alone altered mucin glycosylation, by increasing the sialo/sulfomucin ratio, altering lectin-binding pattern and overexpressing galβ1,3galnac terminal dimers.^5^ Here, we observed that 2 out of 24 mucin glycans identified were significantly higher in HFD compared with the CT and/or HFD+2’FL groups while others did not reach statistical significance, probably due to the limited number of mice analysed. Notably, we found that among the 10 mucin glycans present in all mice, 8 had a lower prevalence only in HFD or were restored by 2’FL supplementation and 3 were less prevalent in the HFD+2’FL group. The ‘restoration’ of glycans prevalence by 2’FL suggests that the prebiotic treatment can be used to counteract alterations induced by HFD. In healthy humans, MUC2 O-glycosylation is uniform while it is altered in patients with active ulcerative colitis and associated with increased inflammation.^37 38^ A different profile was also observed in human colon cancer, linked to tumour metastatic potential and poor prognosis.^39 40^ Understanding the pattern of mucin glycosylation in patients with obesity and metabolic disorders, and how these could be modulated by nutritional treatments, could be useful in inducing the colonisation of specific bacteria associated with beneficial effects. To date, progress has been impeded by the scarcity of research in humans, which is hampered by the need for invasive methods such as biopsy collection.^41^ Surprisingly, a recent study showed that the mucus structure on freshly excreted faecal pellets was identical to that of the faecal pellets in the corresponding colon tissue, suggesting that faecal-associated mucus may support noninvasive strategies for disease diagnosis in humans.^42^

In addition to proteins and enzymes, lipid mediators also play an important role in regulating energy homeostasis. Among them, the eCB system has been shown to regulate energy, glucose and lipid metabolism, gut barrier function and microbiota–host interactions.^43^ Here, we found that mice receiving 2’FL had significantly lower levels of NAEs and decreased expression of *Abdh4* and *Mgl*. In contrast, mice receiving 2’FL showed increased levels of OG and PG compared with the CT and HFD groups. These bioactive lipids were previously reported to be significantly increased in the colon of mice and in the blood of obese humans treated with *Akkermansia muciniphila*, both exhibiting an improved gut barrier, lower inflammation and improved glucose metabolism.^2 44–46^ In addition, mono-OG has been shown to stimulate GLP-1 secretion and improve glucose metabolism.^47^ Short-chain fatty acids (SCFAs) have also been shown to stimulate the secretion of GLP-1 and PYY. However, we did not find any increase in butyrate, propionate and acetate in the caecal content but rather a significant decrease of several SCFAs and branched SCFA after 2’FL treatment (online supplemental figure 6). These findings indicate that 2’FL could affect the metabolism and the mucus layer by acting through the eCB system.

While in vitro studies have demonstrated that alterations of the mucus layer may be directly mediated by 2’FL, we cannot exclude the possibility that, in vivo, they are part of a more complex system involving the gut microbiota. As a prebiotic compound, 2’FL can be metabolised by gut bacteria, stimulating, therefore, the proliferation of specific bacterial groups. By analysing the gut microbiota composition, we observed significant clustering according to the diet, with 2’FL supplementation inducing the most significant changes. Specifically, CT and HFD-fed mice were dominated by uncultured *Desulfovibrionaceae*, while *Akkermansia* and *Bacteroides* were the main bacterial genera in mice receiving 2’FL supplementation. Both genera have been shown to be able to repurpose their mucin degradation machinery for the breakdown of HMOs, reflecting the structural and compositional similarities between HMOs and mucin oligosaccharides.^48 49^ It is, therefore, plausible that the effects of 2’FL on metabolism and mucus could be mediated by bacteria that were significantly affected by the treatment. For example, *A. muciniphila*, a species belonging to the genus *Akkermansia*, is a mucin-degrading specialist residing and proliferating in the mucus layer and affecting metabolism in mice and humans.^46 50 51^ In mice, *A. muciniphila* was previously reported to counteract HFD-induced obesity and prevent the decrease of mucus layer thickness associated with HFD.^2^ On the other hand, *Bacteroides* spp, like *B. uniformis* and *B. acidifaciens*, have been found to be protective against obesity^52–54^ and are highly enriched in colonic mucus layer where they can use mucin glycans as energy source.^55^ Moreover, WSD-fed rodents display an impaired mucus layer associated with a lower abundance of *Bacteroidetes* 3 and *B. thetaiotaomicron*, increased GC differentiation, increased expression of mucus-related genes and sialylated/sulfated mucins ratio.^56^

Since mice supplemented with 2’FL showed increased mucus production and secretion but no changes in mucus thickness, we next assessed whether 2’FL may have stimulated mucus degradation through enhanced bacterial GH activity. Using in-gel fluorescent ABP labelling, we found increased a-L-fucosidase and a-D-galactosidase activity in 2’FL supplemented mice. The PCA for GHs involved in mucus degradation showed distinct clustering patterns between mice fed HFD+2’FL and those fed CT and HFD diet. Among GHs, two b-D-galactosidases and two a-L-fucosidases, assigned to *Bacteroidales* and *Lachnospiraceae* families, were increased in 2’FL supplemented mice while no differences were observed between CT and HFD groups, where these enzymes were either scarce or absent. Furthermore, we found that 2’FL supplementation significantly increased the levels of b-hexosaminidase and b-*N*-acetylhexosaminidase compared with the CT diet. These results suggest that 2’FL stimulates the production of bacterial GHs involved in mucin glycan degradation, perhaps prompted by their involvement in 2’FL degradation.

Further analysis of the mouse proteome showed that HFD had a profound impact on many proteins participating in metabolic processes. Specifically, HFD upregulated proteins involved in protein digestion and absorption while downregulated those involved in different metabolic pathways, among others. Interestingly, 2’FL supplementation had opposite effects, suggesting its ability to counteract the alteration of metabolic processes induced by the HFD. To gain further insights into the mouse results, we analysed the faecal proteome from obese and lean individuals. We showed that there were significant differences, as previously observed,^57^ with some of these changes being similar between HFD-fed mice and obese humans. In addition to obese and lean individuals, even lifestyle-induced weight loss affects proteome.^57 58^ In other clinical contexts, faecal proteome has been used to discriminate patients with adenomas and colorectal cancer and novel stool biomarkers have been proposed for early detection, aiming at reducing their incidence and mortality.^59–61^ This methodology may be used to define new clinical biomarkers capable of detecting the onset of metabolic disorders, enabling their prevention and monitoring the effectiveness of prebiotic/probiotic treatments or personalised dietary interventions in humans for improving individualised patient care and public health outcomes.

In conclusion, our study demonstrates that 2’FL supplementation in the context of HFD-feeding can counteract obesity and metabolic alterations and it is associated with alterations in the intestinal mucus layer through increased expression of secreted and transmembrane mucins, glycosyltransferases and alterations in mucin glycans composition. These changes were accompanied with different profiles of gut microbiota, faecal proteome and eCB system. Our findings suggest that 2’FL has the potential to improve metabolic outcomes in overweight/obese individuals and highlights the importance of investigating the interaction between mucus and gut microbiota. Together these data pave the way for further research on novel strategies and targets for the prevention and/or treatment of obesity and related disorders.

10.1136/gutjnl-2023-330301.supp11 Supplementary data

## Data Availability

All data relevant to the study are included in the article or uploaded as online supplemental information.

## Data availability statement

All data generated or analysed during this study are included in this published article and its online supplemental information files. The raw amplicon sequencing data analysed in this study have been deposited in the European Nucleotide Archive (ENA) at EMBL-EBI under accession number PRJEB72192. The mass spectrometry proteomics data have been deposited to the ProteomeXchange Consortium via the PRIDE [62] partner repository with the dataset identifier PXD049406.
