# Supplementary material for: Human milk oligosaccharide 2’-fucosyllactose protects against high-fat diet-induced obesity by changing intestinal mucus production, composition and degradation linked to changes in gut microbiota and faecal proteome profiles in mice
Source: Gut. 2024 May 13;73(10):1632–49. doi: 10.1136/gutjnl-2023-330301 (PMC11420753; doi:10.1136/gutjnl-2023-330301)
Supplement: Supplementary data [file gutjnl-2023-330301supp002.pdf]

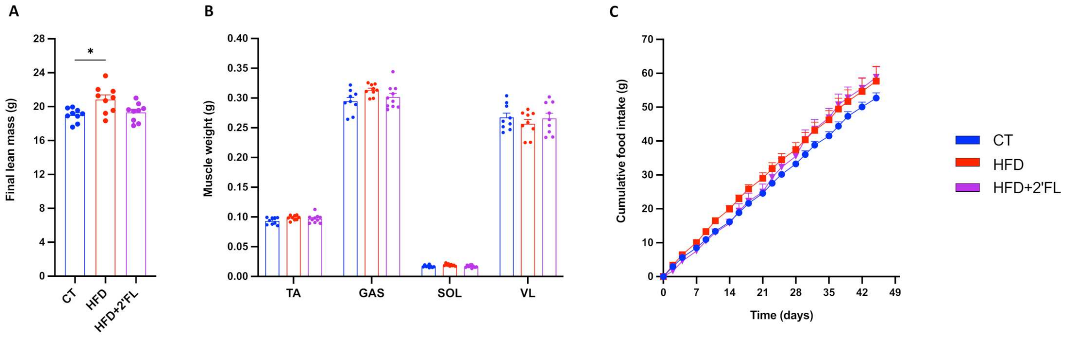

Supplemental Figure 1. (A) Final lean mass and (B) muscle weights (TA = tibialis anterior, VL = vastus lateralis, GAS = gastrocnemius, SOL = soleus). (C) Cumulative food intake. Data are means±s.e.m (n= 9-10/group). Data were analysed using one-way ANOVA for A and B and according to two-way ANOVA for C followed by Tukey post hoc test. *P < 0.05;

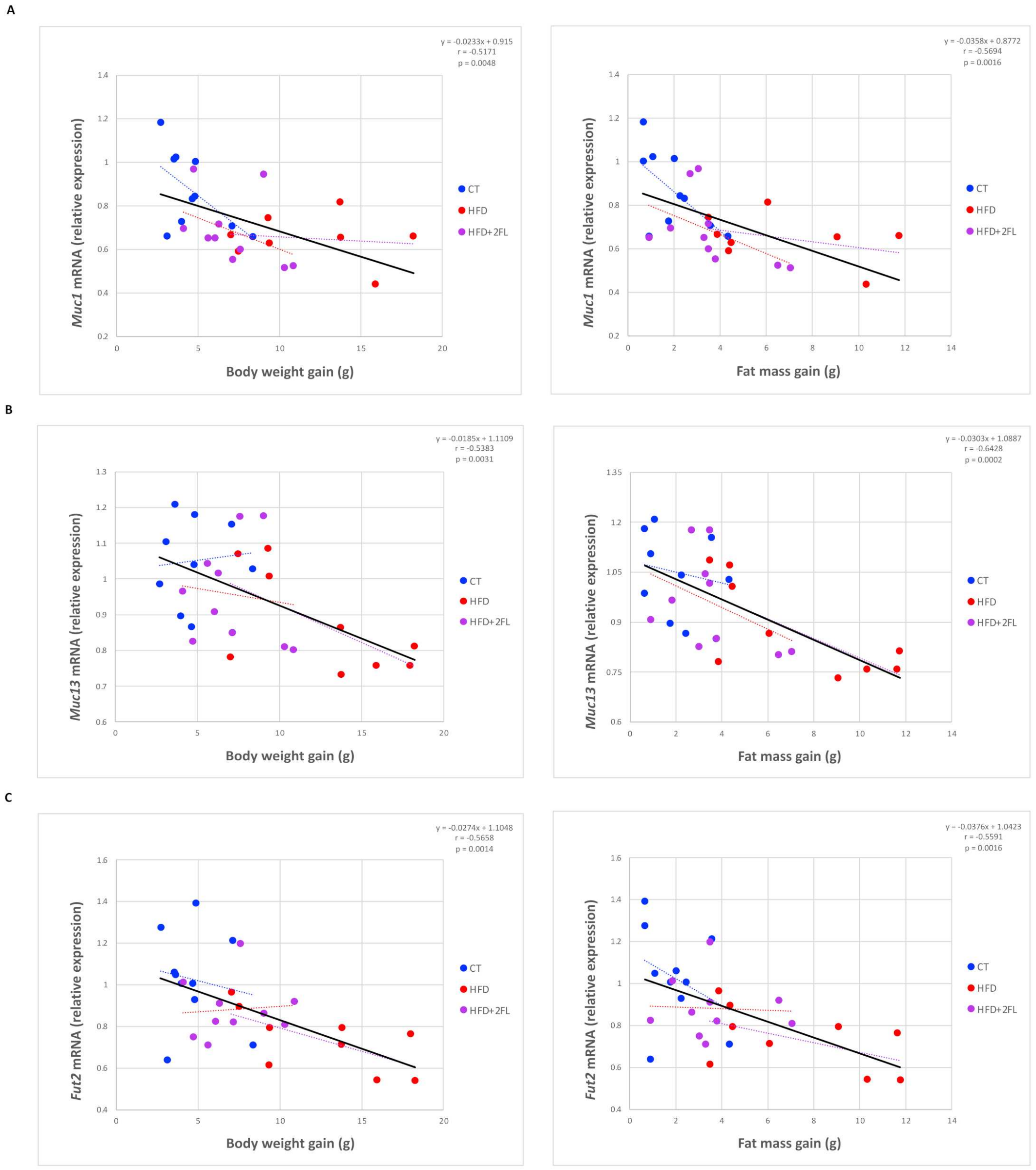

Supplemental Figure 2. Pearson correlation between mRNA colonic expression of (A) *Muc1*, (B) *Muc13* and (C) *Fut2* and body weight gain/fat mass gain (n=9-10/group).

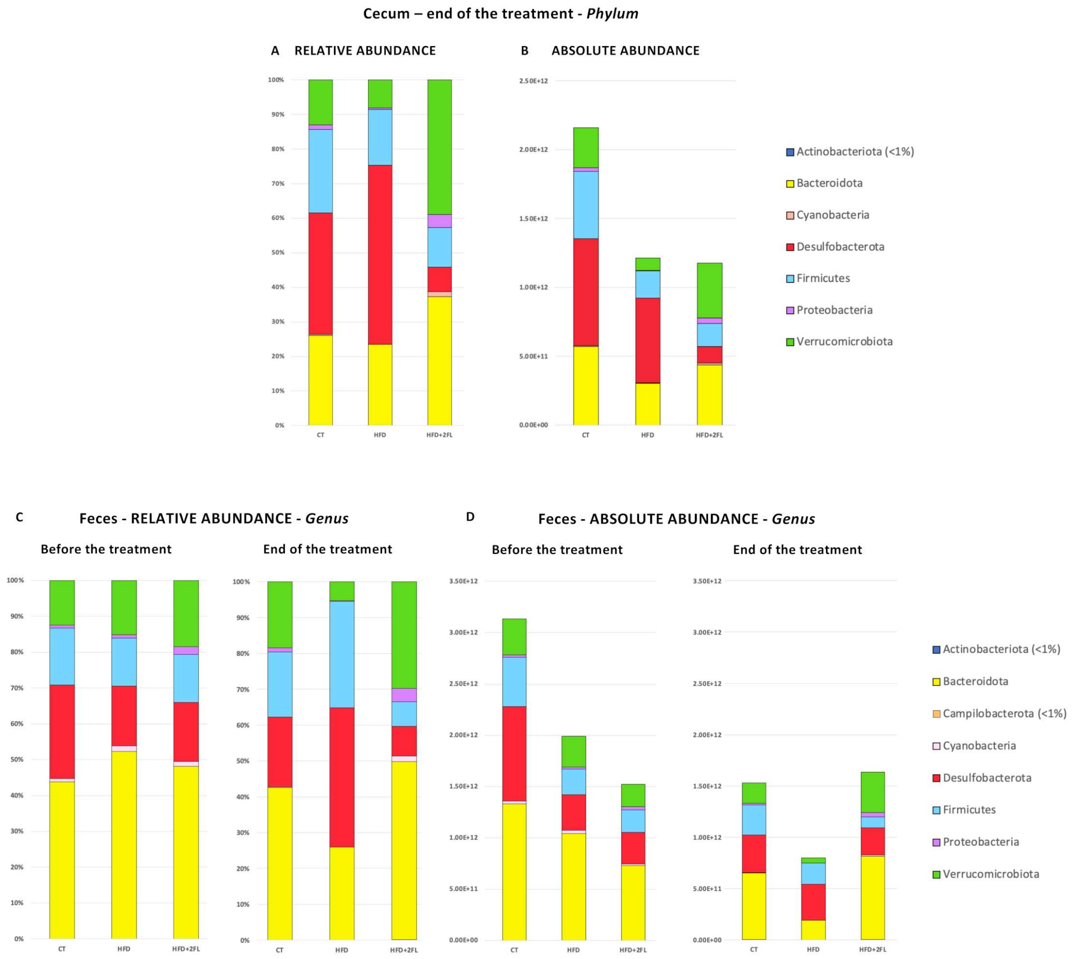

Supplemental Figure 3. (A-D) Bar graphs showing grouped taxonomic profiles of the gut bacteria at a phylum level: (A,B) relative and absolute abundance in the cecum, before and at the end of the treatment; (C,D) relative and absolute abundance in the feces, at the end of the treatment. (n= 9-10/group).

Functional Annotation Clustering

HFD vs CT

▲ UPREGULATED

Annotation Cluster	Enrichment Score	Term
1	9.4	**Proteolysis**<br>**Protease**<br>**Peptidase activity**<br>**Hydrolase**<br>**Hydrolase activity**
2	5.8	Extracellular space<br>Disulfide bond<br>Extracellular region
3	5.1	**Metallopeptidase activity**<br>**Aminopeptidase activity**<br>**Aminopeptidase**
4	3.8	**Peptidase S1**<br>**Peptidase S1, trypsin family, active site**<br>**Protein digestion and absorption**<br>**Serine-type peptidase activity**<br>**Trypsin-like cysteine/serine peptidase domain**<br>**Activation peptide**<br>**Charge relay system**<br>**Serine-type endopeptidase activity**<br>**Tryp_SPc**<br>**Serine protease**<br>**Peptidase S1A, chymotrypsin-type**<br>**Zymogen**
5	3.5	Metalloprotease<br>Metallopeptidase activity<br>Zinc ion binding<br>Peptide catabolic process<br>Metal ion binding<br>Metal-binding<br>Zinc
6	2.8	CUB 1<br>CUB 2<br>CUB domain<br>CUB
7	2.6	ZP<br>Zymogen granule membrane<br>Zona pellucida domain<br>Cytoplasmic vesicle

▼ DOWNREGULATED

Annotation Cluster	Enrichment Score	Term
1	4.7	**Carbon metabolism**<br>**Biosynthesis of amino acids**<br>**2-Oxocarboxylic acid metabolism**
2	3.7	Extracellular region<br>Secreted<br>Extracellular space<br>Signal<br>Disulfide bond<br>Glycoprotein
3	3.3	**Carbon metabolism**<br>**Metabolic pathways**<br>**Tricarboxylic acid cycle**<br>**Citrate cycle (TCA cycle)**<br>**Glyoxylate and dicarboxylate metabolism**<br>**Mitochondrion**
4	2.3	**Lipid metabolism**<br>**Glyoxylate and dicarboxylate metabolism**<br>**Lipid metabolic process**<br>**Lipid catabolic process**
5	2.3	Pancreatic secretion<br>Hydrolase<br>Hydrolase activity
6	1.9	**NAD binding**<br>**Oxidoreductase activity**<br>**Oxidoreductase**<br>**Proton acceptor**<br>**NAD**<br>**Mitochondrion**
7	1.3	Calcium<br>Metal ion binding<br>Metal-binding

HFD+2'FL vs HFD

▲ UPREGULATED

Annotation Cluster	Enrichment Score	Term
1	4.8	Extracellular space<br>Extracellular region<br>Secreted<br>Signal<br>Disulfide bond
2	3.4	**Carbon metabolism**<br>**Glycolysis / Gluconeogenesis**<br>**Biosynthesis of amino acids**<br>**Canonical glycolysis**<br>**Glycolysis**<br>**Glycolytic process**<br>**Hydroxylation**
3	2.3	**Oxidoreductase activity**<br>**Oxidoreductase**<br>**NAD binding**<br>**Proton acceptor**<br>**NAD**
4	2.2	**Lipid metabolic process**<br>**Lipid metabolism**<br>**Lipid catabolic process**
5	1.7	Myelin sheath<br>Catalytic activity<br>ADP binding<br>Membrane raft<br>Nucleotide binding<br>Methylation<br>ATP binding<br>Phosphorylation<br>Kinase activity<br>Kinase<br>Transferase activity<br>Transferase<br>ATP-binding<br>Nucleotide-binding<br>Nucleus
6	1.7	Cytosol<br>Acetylation<br>Cytoplasm
7	1.6	**Glycolysis / Gluconeogenesis**<br>**Glycyl lysine isopeptide (Lys-Gly)**<br>**Isopeptide bond**<br>**Ubl conjugation**
8	1.6	Antimicrobial<br>Inflammatory response
9	1.3	**Mitochondrion**<br>**Transit peptide**<br>**Mitochondrial matrix**<br>**Mitochondrial inner membrane**

▼ DOWNREGULATED

Annotation Cluster	Enrichment Score	Term
1	4.0	**Protease**<br>**Proteolysis**<br>**Peptidase activity**<br>**Aminopeptidase**<br>**Aminopeptidase activity**<br>**Hydrolase**<br>**Metalloprotease**<br>**Metallopeptidase activity**<br>**Peptide catabolic process**<br>**Hydrolase activity**<br>**Zinc ion binding**<br>**Metal ion binding**<br>**Metal-binding**<br>**Zinc**
2	2.9	CUB 1<br>CUB 2<br>CUB domain<br>CUB
3	2.6	ZP<br>Zymogen granule membrane<br>Zona pellucida domain<br>ZP<br>Cytoplasmic vesicle
4	2.4	Extracellular space<br>Extracellular region<br>Secreted
5	2.1	Cadherin 4<br>Cadherin 3<br>Cadherin 1<br>Cadherin 2<br>Cadherin<br>Calcium<br>Cadherin conserved site<br>Cadherin<br>Cadherin-like<br>Homophilic cell adhesion<br>CA<br>Cell adhesion<br>Integral component of plasma membrane
6	1.9	**Protein digestion and absorption**<br>**Peptidase S1**<br>**Peptidase S1, trypsin family, active site**<br>**Peptidase S1A, chymotrypsin-type**<br>**Trypsin-like cysteine/serine peptidase domain**<br>**Pancreatic secretion**<br>**Serine-type peptidase activity**<br>**Serine protease**<br>**Tryp_SPc**<br>**Serine-type endopeptidase activity**

Supplemental Figure 4. Functional annotation clustering performed with DAVID, showing annotation clustering, enrichment scores and terms significantly up/down-regulated by HFD and HFD+2’FL in mice. Only annotation clusters with enrichment scores ≥ 1.3 (corresponding to P-values <0.05) are shown. Terms that changed in an opposite way in HFD-fed mice compared to HFD+2’FL mice are highlighted in bold and red/blue.

Functional Annotation Clustering

Obese vs Normal subjects		
▲ UPREGULATED		
**Annotation Cluster**	**Enrichment Score**	**Term**
1	3.0	carboxypeptidase activity<br>ACT_SITE:Proton donor/acceptor<br>**proteolysis**<br>**Metalloprotease**<br>Carboxypeptidase<br>**Protease**<br>**zinc ion binding**<br>Metal-binding<br>**Zinc**
2	2.6	anchored component of membrane<br>GPI-anchor<br>LIPID:GPI-anchor amidated serine<br>PROPEP:Removed in mature form<br>Lipoprotein
3	2.6	DOMAIN:P-type 1<br>DOMAIN:P-type 2<br>alpha-1,4-glucosidase activity<br>Starch and sucrose metabolism<br>**Glycoside hydrolase, family 31**<br>P-type trefoil<br>PD<br>Galactose mutarotase-like domain<br>Glycosidase<br>Glycosyl hydrolase, family 13, all-beta<br>**hydrolase activity, hydrolyzing O-glycosyl compounds**<br>Galactose metabolism<br>**Glycoside hydrolase, superfamily**<br>Carbohydrate digestion and absorption<br>Sulfation<br>Metabolic pathways<br>Signal-anchor<br>carbohydrate binding<br>apical plasma membrane<br>Helical; Signal-anchor for type II membrane protein<br>Lumenal<br>**Cytoplasmic**<br>integral component of membrane<br>Repeat<br>Helical<br>Extracellular<br>Transmembrane helix<br>Transmembrane<br>Disordered
4	2.5	**proteolysis**<br>**Activation peptide**<br>**Protease**<br>Pancreatic secretion<br>**Protein digestion and absorption**<br>**Zymogen**
5	1.9	N-linked (GlcNAc...) asparagine<br>Glycoprotein<br>Cell membrane<br>membrane<br>plasma membrane<br>Membrane

Supplemental Figure 5. Functional annotation clustering performed with DAVID, showing annotation clustering, enrichment scores and terms significantly upregulated in obese human subjects compared to normal ones. Only annotation clusters with enrichment scores ≥ 1.3 (corresponding to P-values <0.05) are shown. Terms that are similar to those enriched in HFD-fed mice are in red and some of the terms related to metabolism are highlighted in light red.

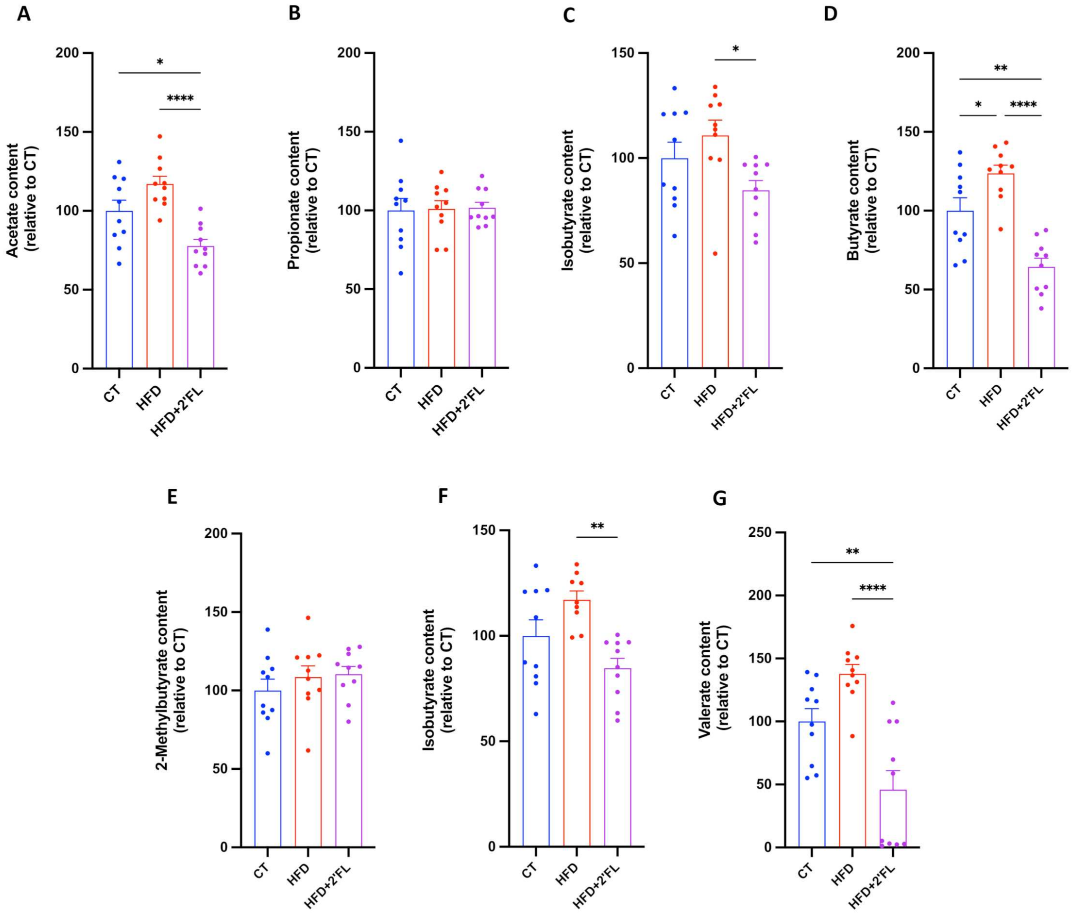

Supplemental Figure 6. Short-chain fatty acids (SCFAs) content in the cecal content. (A) acetate, (B) propionate, (C) isobutyrate, (D) butyrate, (E) 2-methylbutyrate, (F) isovalerate, (G) valerate. Data are means±s.e.m (n= 11-12/group). Data were analysed using one-way ANOVA followed by Tukey post hoc test. *P < 0.05; **P < 0.01; ***P < 0.001; ****P < 0.0001.

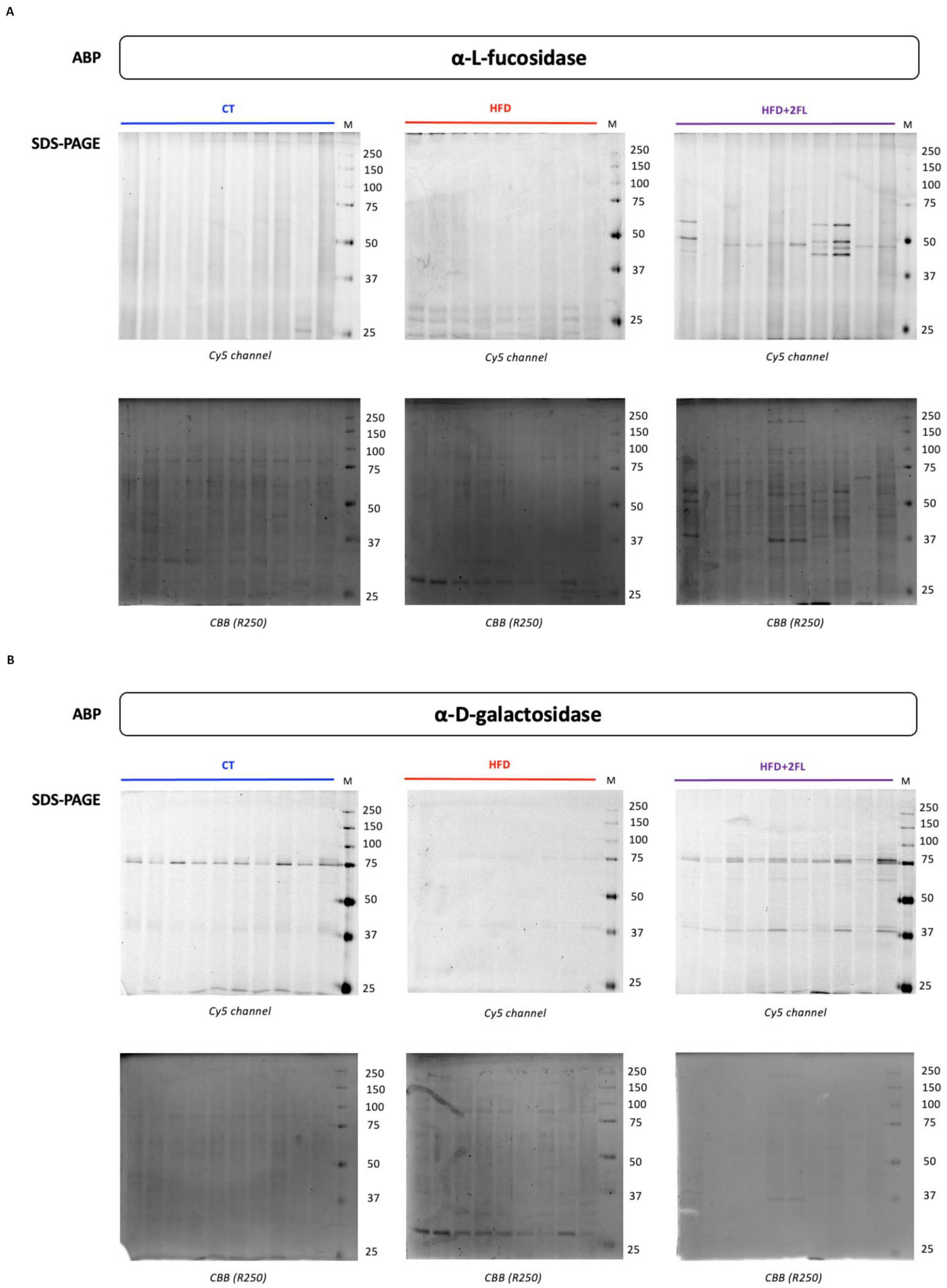

Supplemental Figure 7. In-gel fluorescent ABP labelling. (A) 1 µM for alpha-L-fucosidase labeling (JJB38 1) and (B) 0.5 µM for alpha-D-galactosidase (TB474) and their relative Coomassie Brilliant Blue (CBB) staining (n=9-10/group).
