# Supplementary material for: Human milk oligosaccharide 2’-fucosyllactose protects against high-fat diet-induced obesity by changing intestinal mucus production, composition and degradation linked to changes in gut microbiota and faecal proteome profiles in mice
Source: Gut. 2024 May 13;73(10):1632–49. doi: 10.1136/gutjnl-2023-330301 (PMC11420753; doi:10.1136/gutjnl-2023-330301)
Supplement: Supplementary data [file gutjnl-2023-330301supp001.pdf]

MATERIALS AND METHODS

Mice and diets

Seven-week-old male C57BL/6J mice (Janvier, Le Genest-Saint-Isle, France) were co-housed in pairs under Specific and Opportunistic Pathogen Free conditions (SOPF) in a controlled environment (temperature of 22 ± 2 °C, 12-h daylight cycle) with free access to food and water. Upon arrival, all the mice underwent a 1-week acclimatization period, during which they were fed a control diet [1] (AIN93Mi, Research Diet, New Brunswick, NJ, USA).

A set of 30 mice was randomly divided into 3 groups of 12 mice: 1) CT group, fed a control diet 2) HFD group, fed a high-fat diet (60% fat and 20% carbohydrates (kcal/100g), D12492, Research diet, New Brunswick, NJ, USA), and 3) HFD+2'FL group, fed a HFD diet supplemented with 10% of prebiotic 2'-fucosyllactose added in drinking water (DSM, Denmark). The dose of 10% of 2'FL represents the effective dose to elicit metabolic effects.[2,3] The treatment continued for 6 weeks.

Body weight, food and water intake were recorded three times per week. Body composition was assessed once a week by using a 7.5-MHz time-domain nuclear magnetic resonance (LF50 minispec; Bruker, Rheinstetten, Germany). Feces were harvested weekly since the beginning (Day 0), until the end of the experiment (Day 45). All mouse experiments were approved by and performed in accordance with the guidelines of the local ethics committee. Housing conditions were specified by the Belgian Law of 29 May 2013, regarding the protection of laboratory animals (agreement number LA1230314).

Oral Glucose Tolerance Test

One week before the end of experiment, the mice were fasted for 6 hours before receiving an oral gavage glucose load (2 g glucose per kg body weight). Blood glucose was measured 30 minutes before (time point -30), just prior the oral glucose load (time point 0) and then after 15, 30, 60, 90 and 120 minutes. Blood glucose was determined with a glucose meter (Accu Check, Roche, Switzerland) on blood samples collected from the tip of the tail vein.

Tissue sampling

At the end of the experiment (week 6) and after 6h of fasting, all mice were anesthetized with isoflurane (Forene®, Abbott, Queenborough, Kent, England) and blood was collected from the

portal and cava veins. Then, the mice were immediately euthanized by cervical dislocation. Adipose depots (epididymal, subcutaneous, visceral and brown), muscles (tibialis anterior, vastus lateralis, gastrocnemius, soleus) and intestinal segments (jejunum, ileum, caecum and colon) were dissected, weighed and immersed in liquid nitrogen before long-term storage at −80 °C for further analysis.

One segment of colon from each mouse was opened, without flushing it before, for the collection of the mucus layer by gently scraping with a microscope glass slide and then weighed.

Biochemical Analysis

To determine the plasma insulin concentration, blood was harvested from the tip of the tail vein using capillaries prior to glucose load (−30 min) and 15 min after glucose load. Plasma insulin concentration was measured using an ELISA kit (Mercodia, Uppsala, Sweden), according to the manufacturer's instructions. Insulin resistance index was determined by multiplying the area under the curve of the blood glucose (−30 to 15 min) and plasma insulin (-30 min and 15 min).

Plasma Multiplex Analysis

Plasma levels of glucagon-like peptide 1 (GLP-1), peptide YY (PYY), ghrelin, leptin and glucagon were measured from the portal vein by multiplex assay kits based on chemiluminescence detection and following manufacturer's instructions (Meso Scale Discovery (MSD), Gaithersburg, MD, USA). Analyses were performed using a QuickPlex SQ 120 instrument (MSD) and DISCOVERY WORKBENCH® 4.0 software (MSD, Rockville, MD, USA).

RNA Preparation and gene expression analysis by real-time qPCR analysis

Total RNA was prepared from tissues using TriPure reagent (Roche). Quantification and integrity analysis of total RNA was performed by running 1 µl of each sample on an Agilent 2100 Bioanalyzer (Agilent RNA 6000 Nano Kit, Agilent). cDNA was prepared by reverse transcription of 1 µg total RNA using a Reverse Transcription System kit (Promega, Leiden, The Netherlands). Real-time PCRs were performed with the StepOnePlus real-time PCR system and software (Applied Biosystems, Den Ijssel, The Netherlands) using Mesa Fast qPCR sybr green mix (Eurogentec, Seraing, Belgium) and with the CFX Manager 3.1 software (Bio-

Rad, Hercules, CA) using Mesa Fast qPCR (GoTaq qPCR Master Mix, Promega, Madison, WI, USA) for detection, according to the manufacturer's instructions. RPL19 was chosen as housekeeping gene. All samples were run in duplicate in a single 96-well reaction plate, and data were analyzed according to the 2- ΔΔCt method. The identity and purity of the amplified product was checked through analysis of the melting curve carried out at the end of amplification. Primer sequences for the targeted mouse genes are available in Supplemental Table 9.

Analysis of the mucus layer thickness, goblet cells and immunohistochemistry

Colon segments were immediately removed and fixed in Carnoy's solution (ethanol 6: acid acetic 3: chloroform 1, vol/vol) for 2h at 4 °C. They were then immersed in ethanol 100% for 24 h. For the analysis of the mucus layer thickness and goblet cells, paraffin sections of 5 µm were stained with alcian blue. Images were captured at × 20 magnification and obtained using a SNC400 slide scanner and digital Image Hub software 561 (Leica Biosystems, Wetzlar, Germany). Analyses were performed using ImageJ (version 1.48r, National Institutes of Health, Bethesda, Maryland, USA) in a blinded manner. For the mucus layer thickness, two to six fields were used for each mouse and a minimum of 20 different measurements were made perpendicular to the inner mucus layer per field. For the goblet cells, the luminal side, muscularis mucosae, submucosa and muscle layer were removed and the blue area and the total area were measured separately in the remaining mucosal part of the colon. The proportion of the goblet cells was quantified based on the ratio between the blue area over the total area.

Histology and Fluorescent in situ hybridization

Segments of the distal colon from mice were fixed in water-free Methanol-Carnoy's fixative [60% methanol, 10% chloroform and 30% acetic acid] before paraffin embedding. Paraffin sections were dewaxed with Xylene substitute and hybridized with a general bacterial probe, EUB 338 conjugated to C3 (Merck, Ref: MBD0033). Immunostaining after hybridizations was performed with anti-MUC2C3 antiserum as described previously [4]. Pictures were obtained with a LSM800 confocal microscope from Zeiss.

Bacterial distance and density

The bacterial penetration of the mucus was assessed using two parameters: the distance from the bacterial front to the epithelial cells and the density of bacterial cells within the inner mucus layer. The location of the bacterial front was easily delineated as the outermost border of the zone with high intensity for bacterial stain. The inner mucus was defined as the MUC2 positive layer between the bacterial front and the epithelial cells. To assess the first parameter, at least 10 pictures from different locations of at least 2 different distant sections were analyzed per mouse, with at least 10 measurements (distance between bacterial front and closest epithelial cell) taken per pictures to determine the average distance between the bacterial front and the apical side of the epithelial cells. For the second parameter, the bacterial density of the inner mucus, the area of the MUC2 positive layer between the bacterial front and the epithelial cells was measured and bacteria within this layer were counted manually by two independent investigators in a blinded manner. For this analysis, at least 5 pictures were analyzed per mouse. Analyses were performed using (Fiji Is Just) ImageJ 2.14.0/1.54f For Mac OS and 2.14.0 for Windows. Measurements were first averaged per section, then per mouse, then per group.

Mucin glycan extraction and composition

Colonic mucus was suspended in 400 µl mucin extraction buffer (0.2 M Tris, pH 8, 1% SDS, 10 mM DTT). The samples were incubated at 60°C for 90 min. Iodoacetamide was added from a 1M stock solution to a final concentration of 100 mM. The samples were incubated at RT for 90 min in dark. The reduced samples were spin filtered through a 100k MWCO amicon 0.5 filter (merck) for 15 min at 14000 g. Lithium dodecyl sulphate (LDS) loading buffer (10 µl; Thermo Fischer) was added to the samples and loaded onto a 1% vertical agarose gel cast in Tris-Glycine-SDS (TGS) buffer (Biorad). Vertical agarose gel electrophoresis (VAGE) was carried out at 100 V for 45 min. The mucins/proteins were transferred onto Immobilon Psq(Merck) in Tris-glycine [5] buffer, using Trans-blot Turbo (25 V, 1 A, 60 min; Biorad). The region of the blot were mucins migrated was cut out and the blot was immersed into 500 µl 0.5M $NaBH_4$ in 0.05 M NaOH. The β-elimination reactions were incubated at 45°C for 16 h and quenched by the stepwise addition of 1ml 5% aqueous acetic acid. The samples were desalted on in-house prepared cation exchange columns using Amberlite 50Wx8 H+ 200-400mesh. The

BMJ Publishing Group Limited (BMJ) disclaims all liability and responsibility arising from any reliance placed on this supplemental material which has been supplied by the author(s)

samples were dried under vacuum and removal of borates was carried out with co-evaporation with methanol under nitrogen.

For the base required for permethylation, 400 µl of 50% NaOH were mixed with 800 µl dry MeOH and 4 ml of anhydrous DMSO. The resulting gel was washed 5 times with 4 ml DMSO before resuspended in 4 ml DMSO. The dried samples were dissolved in 100 µl anhydrous DMSO, followed by the addition of 150 µl of the prepared base and 75µl of iodomethane. The samples were vortexed for 2 h at 2000 rpm and the reactions were quenched by the addition of 500 µl H2O. Excess of iodomethane was removed with a flow of nitrogen.

The permethylated glycans were loaded onto a Swift-HLB cartridge (Merck). Salts and other hydrophilic contaminants were removed with 4x1 ml washes with H2O and permethylated glycans were eluted with 4x1 ml of MeOH. The eluted glycans were dried under vacuum and redissolved in 10 µl of 30% acetonitrile in 0.1% aqueous trifluoroacetic acid (TA30). The sample (0.5 µl) was mixed with 0.5 µl of 2,5-dehydroxy-benzoic acid (DHB, 20 mg/ml in TA30) and spotted onto a MTP ground steel MALDI target plate. The samples were analysed by MALDI-ToF MS on a Bruker Autoflex in positive reflectron mode. Peak detection and integration in the mass spectra was done using flexAnalysis (v3.4, Bruker Daltonics) with the following settings: Peak detection algorithm was Snap2, signal to noise threshold = 2, relative intensity threshold = 0, minimum intensity threshold = 2, SNAP2 average composition was set to “sugar”, baseline subtraction was set to TopHat. Relative abundance of each peak identified as glycan was calculated as the area of the peak over the sum of all peaks that were identified as glycans. Only glycans present in at least 3 mice and in at least one group were shown.

Endocannabinoid and lipid content

The endocannabinoid and lipid content in the cecal tissue was analyzed by UHPLC-MS. Briefly, lipids were extracted by ultraturax homogeneisation and internal standards (d_4-AEA, d_4-PEA, d_4-OEA, d_4-SEA and d_5-1-2-AG) were added, followed by protein precipitation (acetone) and recover the supernatant. The samples were analyzed with Xevo-TQS mass spectrometer (from Waters). Absolute quantifications were obtained first by normalizing the area under the curve [6] of the lipid species with the AUC of the respective internal standard and second by extrapolation of the compound’s ratio in his own calibration curve. The LC-MS methods was the following: BEH LC-18 column 50*2.1, 1.7µm (Waters) at 40°C. The mobile phase consisted

BMJ Publishing Group Limited (BMJ) disclaims all liability and responsibility arising from any reliance placed on this supplemental material which has been supplied by the author(s)

in a gradient between A: H_2O 25% -MeOH 75%; B: MeOH 100%, all containing acetic acid (0.1%). ESI probe operated in positive mode was also used for sample ionization. The mass spectrometer parameters were the following: capillary voltage: 2.9kV ; cone voltage : 30V ; desolvation temperature : 550°C ; desolvation gas flow : 1100L/Hr : cone gas flow : 170L/Hr : nebuliser : 6bar.

DNA extraction and 16S rRNA gene amplicon sequencing

Analysis of gut microbiota composition was performed for fecal samples collected at the beginning (day 0) and at the end (day 45) of the study and for the caecal content collected and kept frozen at -80°C until use. Genomic DNA was extracted using a QIAamp DNA Stool Mini Kit (Qiagen, Hilden, Germany), according to the manufacturer’s instructions, including a bead-beating step. The V4 region of the bacterial 16S rRNA gene was amplified with the primers 515F(GTGYCAGCMGCCGCGGTAA) and 806R (GGACTACNVGGGTWTCTAAT). Purified amplicons were sequenced using Illumina MiSeq technology following the manufacturer’s guidelines. Sequencing was performed at MR DNA (www.mrdnalab.com; Shallowater, TX). Sequences were processed using the QIIME2 pipeline (version 2021.4).[7] Demultiplexed 225-bp paired-end sequences were denoised using DADA2 to obtain an amplicon sequence variant (ASV) table.[8] Singletons (ASV present < 2 times) and ASVs present in less than 10% of the samples were discarded. Taxonomic classification was performed using a pre-trained naive Bayes classifier implemented in QIIME2 against the SILVA 132 reference database.[9] Taxa that could not be identified on genus-level are referred to the highest taxonomic rank identified.

Quantitative PCR for total bacteria

Quantification of total bacteria was carried out by qPCR with universal bacterial primers (338F: ACTCCTACGGGAGGCAGCAG, 518R: ATTACCGCGGCTGCTGG), with the StepOnePlus real-time PCR system and software (Applied Biosystems, Den Ijssel, The Netherlands) using GoTaq qPCR sybr green mix (Promega, Madison, Wisconsin, USA), according to the manufacturer’s instructions. All samples were run in duplicate in a single 96-well reaction plate. The cycle threshold [1] of each sample was compared with a standard curve made by serially diluting genomic DNA isolated from a pure culture of the type strain of *Lactobacillus acidophilus* (DSM 20079 01-21) (BCCM/LMG, Ghent, Belgium; DSMZ, Braunshweig, Germany).

The absolute abundances of individual bacterial genera were estimated by multiplying their relative abundance by total bacterial density as described previously.[10]

Preparation of mouse fecal extracts

Fecal extracts were processed based on the protocol of Redinbo et al.[11], with modifications. Briefly, 1-2 fecal pellets collected at the end of the experiment and stores at -80 °C were rehydrated with 350 µl cold extraction buffer (pH 6.5, 25 mM HEPES, 25 mM NaCl with Roche cOmpleteTM protease inhibitor cocktail). The mixture was then transferred in new tubes containing autoclaved 0.7 mm garnet beads and vortexed to break up dense and fibrous material. Bacterial cells were lysed using MP FastPrep-24™ Classic high-speed benchtop homogenizer (MP Biomedicals, Santa Ana, CA, USA) for 2 minutes at 30 Hertz. The resulting homogenate was sonicated two times for 2 min, with an intermediate step of mixing by inversion. The resulting homogenate was centrifugated at 13,000xg for 10 min at 4 °C and the supernatant was decanted. The total protein concentration was calculated using Pierce™ BCA Protein Assay Kit (#23225, Thermo Fisher Scientific, Waltham, MA, USA). The mouse fecal extract was aliquoted and stored at -80°C until further use.

In-gel activity-based probes (ABP) fluorescent labelling of mouse fecal extracts

Mouse fecal extracts were diluted with buffer (pH 6.5, 125 mM HEPES, 125 mM NaCl, final) to have 1 µg of total protein in 9 µL of lysate working solution. 1 µL of Cy5-ABP at a final concentration of 1 µM for alpha-L-fucosidase labeling (JJB381)[12] and 0.5 µM for alpha-D-galactosidase (TB474)[13] was added to the lysate working solution (9 µL) on ice, and the resulting mixture was incubated at 37 °C for 1 h. The samples were denatured by adding 2.5 µL 5x Laemmli buffer (containing 0.3 M Tris-HCl pH 6.8, 50 % (v/v) 100 % glycerol, 8 % (w/v) dithiothreitol (DTT), 10 % (w/v) sodium dodecyl sulfate (SDS), 0.01 % (w/v) bromophenol blue) and boiled at 98 °C for 5 min. Samples were cooled on ice and run on 1.00 mm 10% polyacrylamide gel at 200 V. Wet-slab gels were scanned for ABP-emitted fluorescence using the Typhoon™ FLA 9500 scanner (Amersham Biosciences, Piscataway, NJ, USA), at 700 PMT and 50 µm resolution. Wet-slab gels were subsequently stained with Coomassie Brilliant Blue (CBB) staining agent to verify accurate protein loading. Full gel images and the relative CBB scanned images can be found in Supplemental Figure 7A,B.

Total proteomic analysis of mouse fecal extracts

5 µg of proteins from mouse fecal extracts were diluted in 5 µL of buffer (pH 6.5, 125 mM HEPES, 125 mM NaCl, final). 100 µL 8 M urea/100 mM ammonium bicarbonate (pH 8) were added to each sample and shacked for 30 minutes, 25 °C, 800 rpm to denature the proteins. Samples were reduced with 10 µL of 20 mM DTT and incubated for 30 minutes at 37 °C and shacked at 800 rpm. The samples were cooled at RT for 10 minutes and then 10 µL 50 mM iodoacetamide (IAA) were added. The samples were incubated in the dark at RT for 30 minutes. 900 µL 20 mM ammonium bicarbonate (pH 8) were added to each sample and then 200 ng of trypsin were added to digest the proteins. The samples were incubated overnight at 37°C and shacked at 500 rpm. The following day, to lower the pH to pH < 3, 10 µL of formic acid (FA) were added. The samples were desalted using stage tips and prepared for LC/MS analysis.

Nano-LC-MS settings for total proteomic analysis

Desalted peptide samples were reconstituted in 30 µL LC-MS solution (97:3:0.1 H_2O, CH_3CN, FA) containing 10 fmol/µL yeast enolase digest (cat. 186002325, Waters) as injection control. Injection amount was titrated using a pooled quality control sample to prevent overloading the nanoLC system and the automatic gain control (AGC) of the QExactive mass spectrometer. The desalted peptides were separated on an UltiMate 3000 RSLCnano system set in a trap-elute configuration with a nanoEase M/Z Symmetry C18 100 Å, 5 µm, 180 µm x 20 mm (Waters) trap column for peptide loading/retention and nanoEase M/Z HSS C18 T3 100 Å, 1.8 µm, 75 µm x 250 mm (Waters) analytical column for peptide separation. The column was kept at 40 °C in a column oven. Samples were injected on the trap column at a flow rate of 15 µL/min for 2 min with 99% mobile phase A (0.1% FA in ULC-MS grade water (Biosolve)), 1% mobile phase B (0.1% FA in ULC-MS grade acetonitrile (Biosolve)) eluent. The 85 min LC method, using mobile phase A and mobile phase B controlled by a flow sensor at 0.3 µL/min with average pressure of 400-500 bar (5500-7000 psi), was programmed as gradient with linear increment to 1% B from 0 to 2 min, 5% B at 5 min, 22% B at 55 min, 40% B at 64 min, 90% B at 65 to 74 min and 1% B at 75 to 85 min. The eluent was introduced by electro-spray ionization (ESI) via the nanoESI source (Thermo) using stainless steel Nano-bore emitters (40 mm, OD 1/32”, ES542, Thermo Scientific). The QExactive HF was operated in positive mode

with data dependent acquisition without the use of lock mass, default charge of 2+ and external calibration with LTQ Velos ESI positive ion calibration solution (88323, Pierce, Thermo) every 5 days to less than 2 ppm. The tune file for the survey scan was set to scan range of 350 – 1400 m/z, 120,000 resolution (m/z 200), 1 microscan, automatic gain control (AGC) of 3e6, max injection time of 100 ms, no sheath, aux or sweep gas, spray voltage ranging from 1.7 to 3.0 kV, capillary temp of 250 °C and an S-lens value of 80. For the 10 data dependent MS/MS events the loop count was set to 10 and the general settings were resolution to 15,000, AGC target 1e5, max IT time 50 ms, isolation window of 1.6 m/z, fixed first mass of 120 m/z and normalized collision energy [5] of 28 eV. For individual peaks the data dependent settings were 1.00e3 for the minimum AGC target yielding an intensity threshold of 2.0e4 that needs to be reached prior of triggering an MS/MS event. No apex trigger was used, unassigned, +1 and charges >+8 were excluded with peptide match mode preferred, isotope exclusion on and dynamic exclusion of 10 sec. In between experiments, routine wash and control runs were done by injecting 5 µl LC-MS solution containing 5 µL of 10 fmol/µL BSA or enolase digest and 1 µL of 10 fmol/µL angiotensin III (Fluka, Thermo)/oxytocin (Merck) to check the performance of the platform on each component (nano-LC, the mass spectrometer (mass calibration/quality of ion selection and fragmentation) and the search engine).

MaxQuant processing

Raw files were analyzed with MaxQuant (version v2.1.4.0).[14] The following changes were made to the standard settings of MaxQuant: Label-free quantification was enabled with an LFQ minimal ratio count of 1. Match between runs and iBAQ quantification were enabled. Searches were performed against a Uniprot database created by merging reviewed (Swiss-Prot) and unreviewed (TrEMBL) sequences (downloaded the 21st September 2022) from mus musculus (taxonomy_id:10090; 88,023 results), bacterial fucosidase (32,942 results), sialidase (73,638 results), galactosidase (146,928 results) and hexosaminidase (37,896 results). “proteingroups.txt” file was used for further modifications in Perseus (version 2.0.7.0)[15], including logarithmic transformation (log_2) and removal of proteins ‘Only identified by site’, ‘Reverse’, ‘Contaminant’ and identified based on only one peptide. Non-existing LFQ value due to not enough quantifies peptides were substituted with zero. To analyze the abundance of proteins, their label-free quantification (LFQ) intensities were

compared using GraphPad Prism (version 9.4.1 for macOS) and MetaboAnalyst (more details in "Statistical and Bioinformatics Analysis").

Caecal Short Chain Fatty acids analysis

We used a derivatization method prior to UPLC-MS analysis. Briefly, cecal contents (50 - 60 mg wet material) were homogenized in double-distilled water and then sonicated 10 min in an iced water bath. An aliquot of the resulting material (50 µL) was transferred into tubes containing acetonitrile (200 µL) and valproic acid (used as internal standard). Following incubation at -20°C (1h) the samples were centrifuged, and supernatants were transferred into glass tubes for derivatization (1 h, 40 °C) using 3-nitrophenylhyrazine in the presence of EDC and pyridine. Samples were then purified by liquid-liquid extraction using chloroform to remove the remaining reagents. The SFCA-containing samples were then analyzed using a Nexera LC 40X3 coupled to ZenoTOF 7600 instrument (from Shimadzu and Ab Sciex, respectively). The SCFA were analyzed using a Kinetex F5 (150 × 2.1 mm; 1.7 µM) column maintained at 40 °C. A gradient between H2O-ACN-acetic acid (94.9:5:0.1; v/v/v) and ACN-acetic acid (99.9:0.1; v/v) was used to separate the different isomers. For compound ionization, an ESI source operated in positive mode was used. SCIEX OS 3.0 was used for data analysis. The signal (AUC) of the different SCFA was normalized to the signal of the internal standard (valproic acid). SCFA content was normalized to the caecal content weight.

Human fecal proteomics

The MASCOT Generic Format files from previously analyzed human fecal proteomes[16] were used to identify and quantify the proteins. The MS/MS data were processed using Sequest HT search engine within Proteome Discoverer 2.5 SP1 against a human protein database obtained from Uniprot (81.579 entries January 2023) trypsin (RK) was specified as cleavage enzyme allowing up to 2 missed cleavages, 4 modifications per peptide and up to 5 charges. Mass error was set to 10 ppm for precursor ions and 0.6 Da for fragment ions. Oxidation on Met (+15.995 Da), Carbamidomethyl on Cys (+57.021 Da), pyro-Glu formation from Gln or Glu (-17.027 Da or – 18.011 Da respectively), Acetylation (+42.011Da) and Met-loss (-131.040 Da) on protein-terminus were considered as variable modifications. False discovery rate (FDR) was assessed using a target/decoy PSM validator and set to <5%. Relative quantification was

performed by taking the number of PSMs for each protein identified. Before statistical analysis, the proteomic data were filtered to only include proteins having unique peptides ≥ 2 and PSMs ≥ 3.

Statistical and Bioinformatics Analysis

Statistical analyses were performed using GraphPad Prism version 9.4.1 for macOS (GraphPad Software, San Diego, CA, USA) and RStudio version 2022.12.0+353. Data are expressed as the mean ± s.e.m. Comparison between three groups at one time-point was performed by one-way ANOVA followed by Tukey's test for normally distributed data and Kruskal-Wallis followed by Dunn's test for not normally distributed data. Comparison between three groups at different time-points was performed by 2-way repeated measures ANOVA, followed by Tukey's test. The results were considered statistically significant at $P < 0.05$. The presence of outliers was assessed using the Grubbs test.

For the gut microbiota, statistical analysis was performed using the R package mare.[17] To account for the varying sequencing depth, the number of reads per sample was used as an offset in all statistical models. Overall microbiota structure was assessed using principal coordinate analysis (PCoA) on beta diversity computed using the Bray-Curtis dissimilarity, representing the compositional dissimilarity between the samples. Significant differences between groups were tested using nonparametric multivariate analysis of variance (PERMANOVA) (*adonis* in the *vegan* package[18]). Differential abundance testing was performed using the *mare* function "*GroupTest*" with both relative and absolute abundance data fitted in generalized linear models assuming a negative binomial distribution. If the fitted model failed to fulfil model assumptions (primarily heteroscedasticity of the residuals), generalized least squares models were used. P-values were adjusted by the Benjamini-Hochberg method for multiple testing. FDR-adjusted p-values < 0.05 were considered statistically significant. As absolute microbiota measurements have been suggested to better reflect true changes,[19] log2 fold change values of absolute abundances of the significant genera identified by differential abundance testing (FDR-p < 0.05) were further visualized using the ggplot2 package (figure 11) .

Statistical analysis for the mouse proteomics, including non-parametric tests (Wilcoxon rank-sum test), volcano plot and principal component analysis (PCA), were done using MetaboAnalyst (version 5.0)[20]. For human proteomics, t-test was performed after data

BMJ Publishing Group Limited (BMJ) disclaims all liability and responsibility arising from any reliance placed on this supplemental material which has been supplied by the author(s)

normalization (Log transformation (base 10) and auto scaling) using MetaboAnalyst and PCA was computed from scaled data using PCA function in “FactoMineR” package. Differences between clusters were estimated by PERMANOVA test with 999 permutations on Euclidean distance using adonis2 function from “vegan” package. Proteins that were significantly up/down-regulated were used to create a gene list and execute Kyoto Encyclopedia of Genes and Genomics (KEGG) pathway and functional annotation clustering, giving which term/annotation groups were enriched (using DAVID 2021, https://david.ncifcrf.gov).[21,22] Following default settings, only clusters with P-values <0.05 (corresponding to enrichment scores ≥ 1.3) were shown in Supplemental figure 4 and 5. For human fecal proteomes, DAVID tool was also used to investigate the molecular function, biological process, KEGG pathway and diseases, as shown in figure 14.

REFERENCES

1 Nobel YR, Cox LM, Kirigin FF, Bokulich NA, Yamanishi S, Teitler I*, et al.* Metabolic and metagenomic outcomes from early-life pulsed antibiotic treatment. Nature Communications 2015;**6**:7486.

2 Lee S, Goodson M, Vang W, Kalanetra K, Barile D, Raybould H. 2'-fucosyllactose Supplementation Improves Gut-Brain Signaling and Diet-Induced Obese Phenotype and Changes the Gut Microbiota in High Fat-Fed Mice. Nutrients 2020;**12**.

3 Lee S, Goodson ML, Vang W, Rutkowsky J, Kalanetra K, Bhattacharya M*, et al.* Human milk oligosaccharide 2'-fucosyllactose supplementation improves gut barrier function and signaling in the vagal afferent pathway in mice. Food Funct 2021;**12**:8507-21.

4 Johansson ME, Gustafsson JK, Holmen-Larsson J, Jabbar KS, Xia L, Xu H*, et al.* Bacteria penetrate the normally impenetrable inner colon mucus layer in both murine colitis models and patients with ulcerative colitis. Gut 2014;**63**:281-91.

5 Rodriguez J, Hiel S, Neyrinck AM, Le Roy T, Potgens SA, Leyrolle Q*, et al.* Discovery of the gut microbial signature driving the efficacy of prebiotic intervention in obese patients. Gut 2020;**69**:1975-87.

6 Grivennikov SI, Wang K, Mucida D, Stewart CA, Schnabl B, Jauch D*, et al.* Adenoma-linked barrier defects and microbial products drive IL-23/IL-17-mediated tumour growth. Nature 2012;**491**:254-8.

7 Bolyen E, Rideout JR, Dillon MR, Bokulich NA, Abnet CC, Al-Ghalith GA*, et al.* Reproducible, interactive, scalable and extensible microbiome data science using QIIME 2. Nat Biotechnol 2019;**37**:852-7.

8 Callahan BJ, McMurdie PJ, Rosen MJ, Han AW, Johnson AJ, Holmes SP. DADA2: High-resolution sample inference from Illumina amplicon data. Nat Methods 2016;**13**:581-3.

BMJ Publishing Group Limited (BMJ) disclaims all liability and responsibility arising from any reliance placed on this supplemental material which has been supplied by the author(s)

9 Quast C, Pruesse E, Yilmaz P, Gerken J, Schweer T, Yarza P*, et al.* The SILVA ribosomal RNA gene database project: improved data processing and web-based tools. Nucleic Acids Res 2013;**41**:D590-6.

10 Jian C, Luukkonen P, Yki-Järvinen H, Salonen A, Korpela K. Quantitative PCR provides a simple and accessible method for quantitative microbiota profiling. PLoS One 2020;**15**:e0227285.

11 Jariwala PB, Pellock SJ, Goldfarb D, Cloer EW, Artola M, Simpson JB*, et al.* Discovering the Microbial Enzymes Driving Drug Toxicity with Activity-Based Protein Profiling. ACS Chem Biol 2020;**15**:217-25.

12 Jiang J. Activity-based protein profiling of glucosidases, fucosidases and glucuronidases. Doctoral Thesis 2016.

13 Kytidou K, Beekwilder J, Artola M, van Meel E, Wilbers RHP, Moolenaar GF*, et al.* Nicotiana benthamiana α-galactosidase A1.1 can functionally complement human α-galactosidase A deficiency associated with Fabry disease. The Journal of biological chemistry 2018;**293**:10042-58.

14 Tyanova S, Temu T, Cox J. The MaxQuant computational platform for mass spectrometry-based shotgun proteomics. Nat Protoc 2016;**11**:2301-19.

15 Tyanova S, Temu T, Sinitcyn P, Carlson A, Hein MY, Geiger T*, et al.* The Perseus computational platform for comprehensive analysis of (prote)omics data. Nat Methods 2016;**13**:731-40.

16 Kolmeder CA, Ritari J, Verdam FJ, Muth T, Keskitalo S, Varjosalo M*, et al.* Colonic metaproteomic signatures of active bacteria and the host in obesity. Proteomics 2015;**15**:3544-52.

17 Korpela K. mare: Microbiota Analysis in R Easily. R package version 1.0.

18 Jari Oksanen, F. Guillaume Blanchet, Michael Friendly, Roeland Kindt, Pierre Legendre, Dan McGlinn, Peter R. Minchin, R. B. O'Hara, Gavin L. Simpson, Peter Solymos, M. Henry H. Stevens, Eduard Szoecs, and Helene Wagner, vegan, https://CRAN.R-project.org/package=vegan, (accessed 14 June 2021).

19 Maghini DG, Dvorak M, Dahlen A, Roos M, Kuersten S, Bhatt AS. Quantifying bias introduced by sample collection in relative and absolute microbiome measurements. Nature Biotechnology 2023.

20 Pang Z, Zhou G, Ewald J, Chang L, Hacariz O, Basu N*, et al.* Using MetaboAnalyst 5.0 for LC–HRMS spectra processing, multi-omics integration and covariate adjustment of global metabolomics data. Nature Protocols 2022;**17**:1735-61.

21 Huang da W, Sherman BT, Lempicki RA. Systematic and integrative analysis of large gene lists using DAVID bioinformatics resources. Nat Protoc 2009;**4**:44-57.

22 Sherman BT, Hao M, Qiu J, Jiao X, Baseler MW, Lane HC*, et al.* DAVID: a web server for functional enrichment analysis and functional annotation of gene lists (2021 update). Nucleic Acids Res 2022;**50**:W216-21.

Paone P, *et al. Gut* 2024; 73:1632–1649. doi: 10.1136/gutjnl-2023-330301
