# Supplementary material for: Human milk oligosaccharide 2’-fucosyllactose protects against high-fat diet-induced obesity by changing intestinal mucus production, composition and degradation linked to changes in gut microbiota and faecal proteome profiles in mice
Source: Gut. 2024 May 13;73(10):1632–49. doi: 10.1136/gutjnl-2023-330301 (PMC11420753; doi:10.1136/gutjnl-2023-330301)
Supplement: Supplementary data [file gutjnl-2023-330301supp011.pdf]

**Supplemental table 9:** primers sequences used for the RT-qPCR

| *Primers* | Forward sequence | Reverse sequence |
|---|---|---|
| ***RPL19*** | GAAGGTCAAAGGGAATGTGTTCA | CCTGTTGCTCACTTGT |
| ***Lyz1*** | GCCAAGGTCTACAATCGTTGTGAGTTG | CAGTCAGCCAGCTTGACACCACG |
| ***Reg3g*** | CATCCACCTCTGTTGGGTTC | TTCCTGTCCTCCATGATCAAA |
| ***Pla2g2a*** | AAGGATCCCCCAAGGATGCCAC | CAGCCGTTTCTGACAGGAGTTCTGG |
| ***Intectin*** | GCACTATTGCAGAGGTCCGT | GTTGCCCCTGATTCTGCTGG |
| ***Tff3*** | CCCTGGTGCTTCAAACCTCT | GGGATGCTTGCTACCCTTG |
| ***Proglucagon*** | TGGCAGCACGCCCCTTC | GCGCTTCTGTCTGGGA |
| ***Math1*** | CAAGTGTGTCCAGCAGTGTG | TTGAGTTTCTTCAAGGCGGC |
| ***Spdef*** | AGGTGCAATCGATGGTTGTG | AGGGTCTGCTGTGATGTTCA |
| ***Elf3*** | CCTATGAGAAGCTGAGCCGA | ACCTCTTCTTCCTTCCAGCC |
| ***Klf4*** | GTGCCCCGACTAACCGTTG | GTCGTTGAACTCCTCGGTCT |
| ***Hes1*** | CCGGCATTCCAAGCTAGAGA | GGTATTTCCCCAACACGCTC |
| ***Agr2*** | GCCAAAGACACCACAGTCAA | CCATCAAGGGTCTGTTGCTT |
| ***Muc1*** | GACATCTTTCCAACCCAGGACA | AAGAGAGACTGCTACTGCCATTAC |
| ***Muc2*** | ATGCCCACCTCCTCAAAGAC | GTAGTTTCCGTTGGAACAGTGAA |
| ***Muc4*** | CTGTGTCTGAGCTGCCTGTATT | GGGTGTCTGTGTTGATGTTGTTG |
| ***Muc13*** | CCCTCATCCTCATCTTGCTGATT | CTCTGCTCTTCTCCATCCTTCTTT |
| ***Muc17*** | CCGACACATTGCTGCTGAGAAT | GCTGTCGTCTTGGGTGCTATTT |
| ***Gcnt1*** | ACAGATTCAGGCTTCCTGTGATT | GCCAGGTGAGATGCCAGTTTA |
| ***Gcnt4*** | ATGTCCTGCAGTTCCATTGAGG | ATGTCCTGCAGTTCCATTGAGG |
| ***B3gnt6*** | GGCCAGATTCTCCTCTCTCAAAC | CAGTGTCGTGGGACTCTTGAAC |
| ***C1galt1*** | ATGGACACAGTCACCTCAAAGG | GAGGTTCTCAGCAACGTCTATGT |
| ***C1galt1c1*** | TCTCACGTCCAAGCCTCGT | TGTGGCCTAGCATAGTGATCAAG |
| ***Fut1*** | AGAATTCGCTTGCACCACCA | AAGAAGGAGCCGGCAGAGA |
| ***Fut2*** | TGAACTTTCGGCTAAGGTACATCT | GGAAGTGGGCCAGAGGAAAG |
| ***Fut8*** | AGGCGAATGGCTGAGTCTCT | TGGCCTTAACAAGCTGTTCTTCT |
| ***St3gal1*** | GCCCACTATGCCAGACACTT | TCAGCAGAGTCAAACCCAGC |
| ***St3gal3*** | TGCTGCGGTCATGTAGGAAA | CAGCGGAGTCAAGGGAAAGA |
| ***St3gal4*** | GGCTCTGGTCCTTGTTGTTG | TCCCTAGAACGGTTGCCAAAA |
| ***St3gal6*** | CACCCCAAAAGCGCAGATTTATT | CCTGCCTGAAACAGAGTCCAA |
| ***St6galnac2*** | CGGATGTTGTTGCTCGTTGC | AGTCGGCTCTTTCTGTTTTCC |
| ***Retnlb*** | CAAGGAAGCTCTCAGTCGTCAA | CACTAGTGCAGGAGATCGTCTTAG |
| ***Atg5*** | ATGGTTTGAATATGAAGGCACACC | TGATGTTCCAAGGAAGAGCTGAA |
| ***Atg7*** | CTTCCTGAGAGCATCCCTCTAATC | CGGCTCGACACAGATCATCATAG |
| ***Nlrp6*** | CCCGAAATGTCATCTGAGTGTTCT | TTCAGGGCCTCGGAAAGGT |
| ***Fcgbp*** | AACTTTGCCCACTGACCTG | CCACAGCCTCCCTGCACT |
| ***Daglb*** | CTCCACCAGCAACAAGACAA | GCAGTTCTCCACTTCTGCATC |
| ***Dagla*** | CCCTCAAGTGCTTCGCTTAC | GTCTTTGCCCAGAACCACA |
| ***Napepld*** | TTCTTTGCTGGGGATACTGG | GCAAGGTCAAAAGGACCAAA |
| ***Abdh4*** | GCACAGGGAAGAAGGTGAAG | AGCATAGACGTGGTGGGATG |
| ***Nat1*** | CCCCGAGTTATCGAGGATTT | CTGCAAGGAACAGAACGATG |
| ***Naaa*** | ATTATGACCATTGGAAGCCTGCA | CGCTCATCACTGTAGTATAAATTGTGTAG |
| ***Faah*** | GTGAGGATTTGTTCCGCTTG | GGAGTGGGCATGGTGTAGTT |
| ***Mgl*** | ATGGTCCTGATTTCACCTCTGGT | TCAACCTCCGACTTGTTCCGAGACA |
| ***Abdh6*** | CTGTCCATAGTGGGGCAAGT | TCAGATGGGTAGTAAGCGGC |
| ***Cb1*** | CTGATGTTCTGGATCGGAGTC | TCTGAGGTGTGAATGATGATGC |
| ***Cb2*** | CTGTGCTGCTCATATGCTGG | GCAGAGCGAATCTCTCCACT |
| ***Gpr119*** | AGCTCTGCTCAGCATACACAG | AAATGCCATCCGAAGGCTAC |
| ***Pparg*** | CTGCTCAAGTATGGTGTCCATGA | TGAGATGAGGACTCCATCTTTATTCA |
